# Strengthening field epidemiology capacity in Canada: a mixed-methods evaluation of the Canadian field epidemiology program

**DOI:** 10.3389/fpubh.2026.1777134

**Published:** 2026-03-20

**Authors:** James A. Flint, Pasha Marcynuk, Anja Bilandzic, Jennifer White, Tambri Housen, David N. Durrheim, Martyn D. Kirk, Kathleen Laberge

**Affiliations:** 1 School of Medicine and Public Health, College of Health, Medicine and Wellbeing, The University of Newcastle, Newcastle, NSW, Australia; 2 Office of Public Health Field Services and Training, Center for Emergency Preparedness, Regulatory, Operations and Emergency Management Branch, Public Health Agency of Canada, Ottawa, ON, Canada; 3 National Centre for Epidemiology and Population Health, College of Law, Governance and Policy, Australian National University, Canberra, NSW, Australia

**Keywords:** Canadian field epidemiology program, evaluation, field epidemiology training program, mixed methods, outbreak response, surveillance, workforce development

## Abstract

**Background:**

Field epidemiology training programs (FETPs) play a critical role in strengthening public health capacity for surveillance, outbreak investigation, and evidence-based decision making. The Canadian Field Epidemiology Program (CFEP), established in 1975, was the first program outside of the United States. This study presents findings from a mixed-methods evaluation examining program outputs, outcomes, and impacts.

**Methods:**

Canadian field epidemiology program was evaluated using a theory-based mixed-methods study design. An online survey was administered to all CFEP graduates completing the program from 2018 to 2023. Semi-structured interviews were conducted with graduates, placement site supervisors, and current managers. Quantitative and qualitative data were analyzed and integrated through triangulation.

**Results:**

The online survey was completed by 24 (69%) CFEP graduates and interviews were conducted with 22 graduates, 15 placement site supervisors, and 13 current managers. Graduates developed and applied technical and non-technical skills across all six CFEP competency domains. Most trainees (91%, *n* = 20) evaluated, enhanced, or implemented a surveillance system; after graduation, 84% (*n* = 16) continued to be involved in surveillance activities. Trainees investigated 145 outbreak investigations, while graduates investigated 48 outbreaks. Trainees and graduates introduced innovations in data management and analysis to support evidence-based decision making. Most graduates (75%, *n* = 18) were employed as epidemiologists after CFEP, primarily at the federal level, with a majority (54%, *n* = 13) progressing to supervisory or management roles. Placement site supervisors and current managers emphasized the contributions of CFEP graduates to strengthening disease surveillance and outbreak response systems, improving data analysis processes, and sharing field epidemiology skills with the broader workforce. Trainees were noted for introducing new analytical tools and approaches, including the use of R and advanced data visualization.

**Discussion:**

CFEP made a substantial contribution to Canada’s public health workforce by developing skilled field epidemiologists who applied their skills to strengthen disease surveillance, outbreak response and evidence-based decision making. Expanding mobilization opportunities, formalizing alumni networks, and providing ongoing professional development opportunities would further strengthen field epidemiology competencies and amplify program impact.

## 1 Background

Field Epidemiology Training Programs (FETPs) strengthen workforce capacity by equipping professionals with skills in disease surveillance, outbreak response, data analysis, and evidence-based decision making. FETPs adopt a work-integrated learning model that embeds trainees within public health agencies where they apply newly acquired skills under the guidance of experienced mentors (1, 2). This model enables trainees to contribute to system strengthening while developing hands-on expertise (3). FETPs aim to build a critical mass of skilled health professionals capable of supporting resilient health systems and responding effectively to emerging and acute public health threats (4).

The FETP model was first implemented by the United States Centers for Disease Control and Prevention (CDC) in 1951 and has subsequently grown into a global multi-partner program (5). There are currently FETPs in 98 countries with more than 20,000 graduates worldwide (1). These programs are connected through regional and global networks, including the Training Programs in Epidemiology and Public Health Interventions Network (TEPHINET) and the Global Field Epidemiology Partnership (GFEP) (1, 6). Field epidemiology training is recognized in the revised International Health Regulations (2005), which recommend that countries maintain a minimum of one trained field epidemiologist per 200,000 population (7).

The Canadian Field Epidemiology Program (CFEP) was established in 1975 and was the first program implemented outside of the United States. CFEP is administered by the Public Health Agency of Canada (PHAC) with the aim of strengthening Canada’s public health system capacity and protecting the health of Canadians (8). The program seeks to achieve this by developing an agile workforce capable of responding to emerging issues in public health, improving public health systems (prevention, preparedness, surveillance and disease control), and enhancing collaboration for timely action nationally and internationally (8). CFEP recruits trainees who have a public health background, focusing on master-level epidemiologists, physicians, and veterinarians. Each cohort consists of around 6 trainees who work as full-time, fixed-term contract employees of the PHAC for the 2-year duration of the program, and are placed within public health departments at national/federal, provincial/territorial, or regional (sub-provincial) government levels. Placement sites may also include non-governmental organizations that provide a population-health mandate, such as humanitarian organizations, as well as Indigenous health organizations. Placement sites provide supervision, mentorship and opportunities for trainees to apply field epidemiology skills. PHAC funds salaries and training costs for each CFEP trainee. As a condition of their PHAC employment, trainees may be mobilized on short notice to support Canadian jurisdictions requesting epidemiological assistance. Trainees may also contribute to epidemiological surge capacity internationally when required (9).

During their 2 years of training, trainees are required to meet eight competency-based deliverables, known as professional experience guidelines (PEGs). These guidelines ensure that trainees obtain comprehensive, hands-on experience in key areas of field epidemiology, preparing them for the challenges of public health leadership and building their public health competencies. The 8 PEGs are: (i) field investigation, (ii) epidemiologic analysis, (iii) public health surveillance system evaluation, enhancement, or implementation, (iv) peer-reviewed publication, (v) public health update, (vi) oral presentation, (vii) general communication, and (viii) public health service (9). Entry into CFEP is competitive, with applicants holding graduate level academic qualifications and having at least 1 year of experience working in public health. In 2023, CFEP commissioned a program evaluation to assess and understand the outcomes and impact of CFEP and identify opportunities for improvement. This paper presents the findings of this mixed methods evaluation.

## 2 Methods

### 2.1 Study aim and key evaluation questions

The aim of the evaluation was to assess outputs, outcomes, and impacts of CFEP and to understand common enablers and barriers to knowledge and skill acquisition and application. The following key questions guided the evaluation:

1. To what extent have CFEP trainees and graduates translated knowledge and skills into public health action?
2. What are the common enablers and barriers to translating knowledge and skills into public health action?
3. To what extent do CFEP trainees and graduates contribute to increased knowledge and skills in the workplace?
4. To what extent have CFEP trainees and graduates contributed to surge response in Canada?
5. To what extent are CFEP graduates prepared to respond to emerging public health issues?
6. Were there any unintended positive or negative consequences of the CFEP experience on trainees?

### 2.2 Study design

This evaluation adopted a theory-based *convergent* mixed-methods design (10). A mixed-methods approach was selected to provide evidence of outputs, outcomes, and impacts of CFEP, as well as the factors that facilitated or constrained these results. An essentialist or realist analytical approach underpinned both the quantitative and qualitative components, assuming that statistical findings reflect underlying real-world phenomena and that qualitative themes derived from explicit or semantic content represent a shared reality rather than socially constructed positions (11). Qualitative and quantitative data were collected within a similar timeframe, analyzed separately, and integrated during interpretation (12, 13). A published FETP impact evaluation framework (Table 1) provided the theoretical basis for guiding the evaluation (14, 15).

**Table 1:** Field epidemiology training program impact evaluation framework.

Level of change	Outputs (product, projects, activities)	Outcomes (short and medium-term effects of program)	Impact (long-term effects of program)
Trainees	*Outputs during the training program* Trainees participate in competency-based Field Epidemiology Training Program and apply skills and knowledge.	*Short term outcomes achieved by trainees during training* Trainees are competent and committed to applying their skills and knowledge in their workplace.	
Graduates	*Individual outputs following graduation* Graduates develop and apply skills to strengthen disease surveillance, investigate outbreaks, conduct operational research and share findings through papers, reports and presentations.	*Short and medium-term outcomes by individual graduates* Skilled graduates strengthen public health activities in their workplace and contribute to a community of practice through alumni networks.	
Public Health System	*Outputs affecting the wider public health system* Graduates embedded across all levels of the public health system, conducting projects and activities that strengthen public health systems. Graduates become junior FETP faculty.	*Short and medium-term outcomes affecting the wider public health system* Experienced Field Epidemiology workforce contributes to strengthening the public health system through routine application of knowledge and skills. FETP graduates support FETP as trainers and mentors.	*Longer-term effects of the program on the wider public health system* Strong public health systems across country. Strong and Sustainable FETP is established.
Community	*Outputs that affect the community* Community based public health activities and outreach programs conducted.	*Short and medium-term outcomes affecting the community* Improved access to higher quality public health services addressing priority community needs; community engaged in public health decision making.	*Longer-term effects of the program on the community* Improved public health realized through reduced morbidity and mortality from communicable and non-communicable diseases.

From Flint JA, et al. (14).

Our approach was guided by Kirkpatrick’s four levels of training evaluation, which assesses a learner’s response to the training (Level 1: reaction), improvements in skills and knowledge (Level 2: learning), application of skills (Level 3: behavior), and the resulting benefits at the organizational level and the broader impacts on the health system (Level 4: results) (16). The evaluation consisted of an online survey administered to all CFEP graduates completing the program between 2018 and 2023, and in-depth interviews with CFEP graduates, their placement supervisors and current managers.

### 2.3 Sampling

All 35 CFEP graduates from 2018 to 2023 were included in the sampling frame for the graduate survey. This timeframe was selected to obtain a current snapshot of the program, have a sufficient sample and include graduates who were in the program pre and post the COVID-19 pandemic. For interviews, graduates were purposefully selected using maximum-variation sampling (17) to ensure representation by (a) level of placement site (national, provincial, territorial, regional, local), (b) gender and (c) year of graduation. A sampling frame of 24 graduates for interview was identified based on these criteria. Team discussions informed saturation, and recruitment continued until no new categories arose from interviews. The placement-site supervisors of graduates interviewed made up the sampling frame for supervisor interviews (*n* = 23), and all current managers nominated by graduates during interview made up the sampling frame for manager interviews (*n* = 17). Managers were ineligible for interview if they had managed the CFEP graduate for less than 3 months. Supervisors and managers were interviewed until data saturation was reached.

### 2.4 Quantitative data collection and analysis

An online survey (Supplementary File 1) was administered to CFEP graduates who completed the program from 2018 to 2023. All graduates were eligible for inclusion, with no exclusion criteria applied. The survey was conducted in English using QuestionPro (2024 version) (18). Graduates were invited to complete the survey by the CFEP Director and were given 3 weeks to complete the survey with two reminder emails sent by the lead evaluator to non-respondents at two-week intervals.

Survey data were de-identified and summarized descriptively using Microsoft Excel (Version 16.98) (19). Respondents and non-respondents were compared to identify any significant differences based on sex, year of graduation, level of work, and location. Analysis was conducted using R (version 4.5.1) (20).

### 2.5 Qualitative data collection and analysis

Graduates were invited to participate in an interview by the CFEP Director. Videoconference or phone interviews were subsequently conducted by an independent bilingual qualitative researcher who did not have any affiliation with CFEP. Graduate interviews lasted approximately 60 min, while supervisor and manager interviews lasted for approximately 30 min. Interviews were conducted in English or French using semi-structured interview guides (Supplementary File 2). All interviews were audio-recorded and transcribed using a professional transcription service. All identifying information was removed from transcripts to maintain participant confidentiality. English transcripts were checked for accuracy by the lead evaluator (JF) and French transcripts by a bilingual researcher (KL).

Transcripts were analyzed thematically using a hybrid inductive-deductive approach described by Braun and Clarke (11) and Proudfoot (21). An essentialist, or realist, method guided the qualitative analysis, focusing analysis on the semantic content of interviews (11). Two researchers (JF, JW), one an epidemiologist and one a social scientist, immersed themselves in the data by reading transcripts several times and noting initial ideas. Both researchers independently coded a subset of transcripts and met to compare codes to ensure conceptual alignment. One researcher (JF) systematically coded the entire dataset, giving full and equal attention to each transcript, with regular cross-checking meetings with the second researcher. NVivo 14 version 14 (release 14.24.3) was used to support analysis (22). Initial analysis was data-driven, using an inductive approach to identify relationships and themes without using a pre-existing framework. These themes were later organized using the FETP impact evaluation framework (14) and Kirkpatrick’s four levels of training evaluation (16). Compelling quotations were identified for potential inclusion in the evaluation report. The primary analyst (JF) has extensive experience in field epidemiology workforce development, and while not having been involved in the design or delivery of the Canadian Field Epidemiology Program (CFEP), his prior work in related FETPs may have shaped his understanding of training models and influenced the interpretation of participant narratives. To mitigate potential bias, analyses were discussed collaboratively with a second researcher with no prior involvement in FETPs. JF also documented potential biases prior to analysis.

### 2.6 Data integration

Following guidance of Fetters et al. (2023), quantitative and qualitative data were integrated during the interpretation phase (23). Findings from the surveys and interviews were examined through a process of triangulation involving weaving, where qualitative findings were used to elaborate quantitative data, and merging, where quantitative and qualitative data were presented in joint displays (24). This approach supported the development of a coherent narrative of the program’s contributions and its alignment with, or divergence from, the program theory (25). Contribution analysis was used to assess the role of CFEP in achieving observed public health outcomes and impacts. Findings from surveys and interviews were integrated to understand program contributions, while acknowledging the role of external factors (26).

## 3 Results

### 3.1 Quantitative

A total of 24 (69%) of the 35 CFEP graduates from 2018 to 2023 completed the online survey, although not all graduates responded to every question in the survey. There were four male and 20 female survey respondents; the response rate was similar for males and females, 67 and 69%, respectively. For the 19 graduates indicating their age category, most (*n* = 16, 84%) were aged 30–39 years with the remaining (*n* = 3, 16%) aged 40–49 years. No graduates identified as a person with a disability or living with a chronic condition. English was the first language for 84% (*n* = 16) graduates, and French for 16% (*n* = 3). Most (*n* = 22, 92%) graduates responding to the survey were enrolled in CFEP’s epidemiology stream, one (4%) was enrolled in the Medical Doctor stream and one (4%) in the Veterinary Medicine stream. Prior to commencing CFEP, graduates reported having worked in the field of public health for an average of 4.7 years (range: 1–10 years). During CFEP, 27% (*n* = 6) of graduates were placed at the Federal level with the Public Health Agency of Canada, 36% (*n* = 8) at the provincial level, and 41% (*n* = 9) at the regional level. Location information was unknown for 1 (4%) graduate.

At the time of interview, all (100%, *n* = 24) graduates were currently employed, with 75% (*n* = 18) working as epidemiologists, 17% (*n* = 4) in managerial/director roles and 8% (*n* = 2) in specialist roles. Of the 23 providing the name of their current employer, most (74%, *n* = 17) were working for the Public Health Agency of Canada. Graduates were also employed by other federal agencies (*n* = 1, 4%), provincial health departments (*n* = 1, 4%), territorial health departments (*n* = 1, 4%), regional health departments (*n* = 2, 9%) and overseas health agencies (*n* = 1, 4%). Graduates filled non-supervisory (46%, *n* = 11), supervisory (38%, *n* = 9) and mid-level managers (17%, *n* = 4) roles.

There were no statistically significant differences between graduates who responded to the survey and those who did not with respect to sex, year of graduation, level of placement (local, provincial, national), and province/territory.

### 3.2 Qualitative

Semi-structured interviews were conducted with 22 CFEP graduates, 15 placement site supervisors, and 13 current managers. Among the CFEP graduates interviewed, 6 (27%) were male and 16 (73%) were female. Of the site supervisors, 4 (27%) were male and 11 (73%) were female. Among the current managers interviewed, 1 (8%) was male and 12 (92%) were female. Graduates, supervisors, and managers represented regional, provincial, and federal levels of government (Table 2).

**Table 2:** Number and percentage of graduate, site supervisors and current managers interviewed by level, Canada, 2024.

Participant group	Public Health Organisation			Total
	Federal	Provincial / Territorial	Regional	
Graduates	7 (32%)	5 (23%)	10 (45%)	22 (100%)
Supervisors	4 (27%)	2 (13%)	9 (60%)	15 (100%)
Managers	5 (38%)	2 (15%)	6 (46%)	13 (100%)
Total	**16 (32%)**	**9 (18%)**	**25 (50%)**	**50 (100%)**

### 3.3 Kirkpatrick level 1: reaction

Most graduates rated their learning experience as good or very good (Table 3). The knowledge of the trainers, the training materials, and delivery of training were the highest-rated components. Supervision at placement sites and by the CFEP supervisor was rated slightly lower.

**Table 3:** Graduate reactions to different components of learning during CFEP training from 2018–2023 (n = 22) among program graduates, 2023.

Training Component	Very good	Good	Acceptable	Poor	Very poor
How the training was delivered	18 (82%)	2 (9%)	2 (9%)	0 (0%)	0 (0%)
The training materials provided	14 (64%)	6 (27%)	2 (9%)	0 (0%)	0 (0%)
The training content	12 (55%)	7 (32%)	3 (14%)	0 (0%)	0 (0%)
The knowledge of the facilitators	17 (77%)	3 (14%)	2 (9%)	0 (0%)	0 (0%)
Support/mentoring during workshops	11 (50%)	8 (36%)	3 (14%)	0 (0%)	0 (0%)
Supervision by the placement site	10 (45%)	4 (18%)	7 (32%)	1 (5%)	0 (0%)
Support/mentoring by CFEP supervisor	11 (50%)	2 (9%)	6 (27%)	2 (9%)	1 (5%)

CFEP graduates offered several suggestions to improve the overall learning experience. With regards to *training content*, several graduates recommended expanding leadership training and including additional modules on advanced analytics, R, GIS, and infectious diseases. One graduate suggested having the ability to personalize training options by selection from a suite of modules offered by the program. In terms of *training delivery*, graduates favored in-person training over online or remote learning. The primary reasons related to improved focus, reduced workplace interruptions, and the relational benefits of learning together: “There are too many distractions with online learning, especially with one’s day job able to intrude… in-person training makes working in time-sensitive and stressful situations much easier; the remote learning opportunities do not generate similar relationship building” (G8). For *placement sites*, graduates recommended expanding the number and type of sites and implementing more rigorous screening to ensure sites have adequate capacity for supervision. Graduates recommended providing clearer guidance on project expectations and suggested mentorship for supervisors to improve consistency in training experiences. *Mobilization* related feedback included providing greater transparency when selecting trainees for mobilizations, increasing national and international mobilization opportunities, and enforcing post-mobilization rest periods to mitigate fatigue and support wellbeing. Graduates also recommended having more opportunities for formal and informal *networking* during and after CFEP.

### 3.4 Kirkpatrick level 2 and 3: learning and behavior

#### 3.4.1 Development and application of Field epidemiology knowledge and skills

Most graduates reported that they were able to apply their knowledge and skills across the professional experience guidelines (Table 4).

**Table 4:** Ability of CFEP trainees from 2018 to 2023 (*n* = 21) to apply field epidemiology knowledge and skills during training, 2023.

During CFEP, I was able to apply my knowledge and skills in:	Strongly agree	Agree	Neither agree nor disagree	Disagree	Strongly disagree	N/A or Do not know
Field investigation	15 (71%)	5 (24%)	0 (0%)	1 (5%)	0 (0%)	0 (0%)
Epidemiologic analysis	8 (38%)	11 (52%)	2 (10%)	0 (0%)	0 (0%)	0 (0%)
Public health surveillance	12 (57%)	8 (38%)	1 (5%)	0 (0%)	0 (0%)	0 (0%)
Peer-reviewed publication	8 (38%)	5 (24%)	6 (29%)	1 (5%)	0 (0%)	1 (5%)
Scientific communication to a variety of audiences	8 (38%)	12 (57%)	0 (0%)	1 (5%)	0 (0%)	0 (0%)
Oral presentations	12 (57%)	7 (33%)	2 (10%)	0 (0%)	0 (0%)	0 (0%)

At the time of the survey, the level of confidence conducting a range of key field epidemiology-related activities reported by graduates is shown in Table 5. Most graduates reported being ‘confident’ or ‘very confident’ in conducting the listed activities. Graduates were slightly less confident leading large-scale, multi-jurisdictional outbreak investigations, implementing analytic studies and conducting analyses from analytic studies.

**Table 5:** Level of confidence of CFEP graduates (*n* = 20) from 2018–2023 conducting key field epidemiology related activities, 2023.

Activity	Very confident^a^	Confident^b^	Not confident^c^
Using surveillance data to guide public health programming	14 (70%)	6 (30%)	0 (0%)
Evaluating a surveillance system and making recommendations for system improvement	16 (80%)	4 (20%)	0 (0%)
Leading a small scale (single jurisdiction) outbreak investigation	14 (50%)	5 (25%)	1 (5%)
Leading a large scale (multi-jurisdictional) outbreak investigation	7 (35%)	10 (50%)	3 (15%)
Being deployed into the field to support an outbreak response	17 (85%)	2 (10%)	1 (5%)
Supporting a public health emergency response within an Incident Management System	13 (65%)	6 (30%)	1 (5%)
Implementing an analytic epidemiologic study (e.g., cohort or case control study)	6 (30%)	12 (60%)	2 (10%)
Conducting descriptive data analysis	18 (90%)	2 (10%)	0 (0%)
Conducting analytic data analysis	11 (55%)	7 (35%)	2 (10%)
Developing evidence-based policy recommendations from data and information	12 (60%)	8 (40%)	0 (0%)
Giving an oral scientific presentation	18 (90%)	2 (10%)	0 (0%)
Writing an abstract for a scientific conference	18 (90%)	2 (10%)	0 (0%)
Writing a scientific manuscript for a peer-reviewed journal	15 (75%)	4 (20%)	1 (5%)
Creating communication products for a non-scientific audience	15 (75%)	5 (25%)	0 (0%)

^aVery confident – I could do this without any support. bConfident – I would need some support. cNot confident – I would need a lot of support.^

#### 3.4.2 Enablers to developing and applying Field epidemiology skills

Graduate interviews identified several enablers supporting the application of field epidemiology skills in the workplace. These enabling factors were organized into the following themes: placement sites, field mobilizations, program reputation and credibility, supportive managers and teams, and professional/alumni networks.

*Placement sites* with clearly defined roles and supportive work cultures were reported as key enablers of skill development during CFEP. Graduates emphasized the importance of supervisors who created learning opportunities, provided expert guidance and enabled access to professional networks and resources. As one graduate reflected, the placement offered a valuable opportunity to engage directly in real-time response efforts, describing the experience as, “very, very hands-on, very close to public health action” (G2).

Outbreak investigations and *field mobilizations* were identified as critical enablers of skill development during training. Trainees reported that experiences working in varied and challenging environments were important for consolidating theoretical knowledge and were, “invaluable for building practical expertise” (G4). One graduate highlighted the valuable integration of knowledge and practice: “CFEP provided a very strong blend of gaining knowledge and then hands-on experience… when we were mobilized, we were able to apply the skills on the ground with a lot of support… that helped build a lot of confidence” (G5).

After completing CFEP, graduates identified the strong *reputation of CFEP* as an enabling factor that generated opportunities to apply field epidemiology skills in professional settings. Participants described how the credibility promoted confidence, and created opportunities to contribute expertise: “The street cred that comes with being a field epi somewhat surprised me. That, I think, gave me the space to be able to step up and provide recommendations and apply some of the knowledge that I might not have otherwise been given the opportunity to” (G19). Similarly, others stated: “The program is held in such high regard that if you come with fresh ideas from the program, people are usually very open to it” (G10), and, “It’s a great thing to have [CFEP] on the resume, people really trust you with whatever role you’re in because they know the quality of that program… it’s like a validity check on your skills” (G6). Graduates reported that the reputation and credibility of CFEP facilitated job and career advancement opportunities. The reputation of CFEP extended beyond Canada, with one graduate mentioning the recognition and trust that came from colleagues at the World Health Organisation (WHO) who had completed similar field epidemiology training programs in Europe.

Graduates also identified *supportive managers and teams* as important enablers of applying CFEP-acquired skills in the workplace. Participants described the value of organizational environments that recognized their expertise and encouraged personal initiative: “The trust of my managers and knowledge of what skills we have as CFEP alumni was definitely something that helped me put these skills into practice” (G16). Another highlighted the importance of autonomy: “One of the really great things about my role is that I have a lot of autonomy to develop my own work plan and projects… I’ve been given a lot of scope to use the skills and tools that I gained through CFEP” (G5).

Both informal (e.g., WhatsApp groups) and formal (e.g., technical working groups) *professional networks* were identified as enabling factors that supported graduates to apply and sustain their field epidemiology skills following CFEP. Graduates described these networks as valuable for providing expert advice, technical resources, and peer support, particularly when dealing with complex or unfamiliar public health challenges. As one graduate explained, “the network is so strong, you really feel supported no matter what you’re doing and no matter what random question comes up, there’s always somebody to help you with it” (G10). The ongoing nature of these professional networks was particularly valued, with another noting, “we have a WhatsApp group for our cohort… we’re five years out, but like every week or every two weeks, somebody will post a question, and somebody will answer it” (G3). These graduate networks were noted for facilitating employment, promotions, and mobilizations.

#### 3.4.3 Barriers to developing and applying Field epidemiology skills

The following themes emerged as barriers to developing and applying field epidemiology skills during and following training: placement sites, field mobilizations, and workplaces.

When there was poor alignment between the priorities of *placement sites* and the interests or expertise of trainees, the number and scope of opportunities to apply and develop skills was limited. Graduates described rigid organizational structures and processes, particularly within government departments, as barriers which inhibited innovation and limited use of the full range of skills gained through CFEP. Poor leadership, frequent supervisor changes, unclear direction, and a narrow scope of work, especially during the COVID-19 pandemic, were also highlighted. Some graduates reported that there was at times a reluctance to sharing opportunities by colleagues at the placement site. Others indicated being overlooked for leadership opportunities due to the temporary nature of placements, limiting their ability to gain experience and apply their skills. The fast pace, high expectations, and burnout experienced during COVID-19 were barriers noted by placement site supervisors.

Graduates reported that COVID-19 travel restrictions substantially reduced the number of *field-mobilization* opportunities available to trainees. The scale and urgency of the pandemic diverted resources and attention from non-COVID-19 outbreaks, reducing opportunities to practice core skills. One graduate reflected: “I feel like an imposter when I still feel green in some aspects of outbreak investigation, because we didn’t really get a lot of traditional outbreak investigation experience” (G14). Graduates noted that limited opportunities to be mobilized for outbreak or emergency response activities following graduation constrained their ability to maintain and strengthen their skills: “One of the issues with the program is that upon graduating from it, the opportunities to get mobilized dry up pretty quickly… it’s tough to continue to exercise those skills and practice those skill sets if you’re not able to be put into the position to do so” (G9).

Graduates perceived resistance to change within organizational cultures and government structures, limiting opportunities to influence decision-making and drive improvements in the *workplace*. One graduate reflected feeling disempowered by the hierarchical systems of the workplace, describing the experience as being like, “a cog in the machine” and being considered too junior to drive change (G13). Another stated that, “sometimes [organizational] priorities and direction don’t align with what you feel from an epi perspective” (G12). Others reported that heavy workloads restricted capacity for in-depth analytical work, even when opportunities were available to apply their skills. Outdated information systems were also identified as barriers to effective surveillance, data management and analysis. In several settings, leadership of outbreak investigations remained reserved for senior staff, limiting opportunities for graduates.

### 3.5 Kirkpatrick level 4: results (outputs, outcomes, impact)

The outputs, outcomes and impacts described by graduates, supervisors, and managers were organized into technical (disease surveillance, outbreak investigation and emergency response, and data analytics) and non-technical themes and are discussed below and summarized in Table 6.

**Table 6:** Integration of qualitative and quantitative data examining outputs, outcomes and impacts from applying skills among CFEP graduates from 2018–2023, Canada, 2024.

Domain	Outputs Graduate Survey	Outcomes / Impacts Supporting Quotations from Graduates	Outcomes / Impacts Supporting Quotations from Placement Site Supervisors	Outcomes / Impacts Supporting Quotations from current managers
Disease surveillance	During CFEP: 91% (*n* = 20) evaluated, enhanced or implemented a surveillance system 45% (*n* = 9) made recommendations to strengthen surveillance that were implemented Following graduation: 84% (*n* = 16) were involved in one or more surveillance activities 74% (*n* = 14) were involved in analyzing surveillance data 63% (*n* = 12) used surveillance data to inform health programming 53% (*n* = 10) improved a surveillance system 47% (*n* = 9) managed a surveillance system 32% (*n* = 6) implemented a new surveillance system 89% (*n* = 17) agreed that CFEP adequately prepared them for supporting surveillance activities	“We basically developed a brand-new COVID-19 surveillance system at the WHO. And skills that I gained from CFEP were used to support my team.” (G6) “I set up our wastewater surveillance system and a data management tool. So for the wastewater surveillance data tool… with the wastewater surveillance, there was definitely some very rapid data to action that we saw, being able to turn around within 24 h to notify the community of the presence of COVID in the wastewater. I think that was probably the most dramatic data to action that we have seen.” (G19) “I participated in multiple updates of national definitions for surveillance, leading working groups [which] ensured the surveillance system captured more accurate and policy-relevant information, ultimately guiding vaccination campaigns and outbreak management at the national level” (G22).	“She was able to help us with an aberration detection surveillance system and the evaluation of it… that made a huge change, before we used to do it in a very cumbersome, like, eyeballing way. And now it’s all automated. So, that made a huge help to the epi team and to the infectious disease program team…before we would not have time to do the weekly detection, but now we do. So, that’s a huge help. That’s a big, significant piece of work that she has helped us with.” (S14) “She helped create some procedures and policies around…wastewater surveillance. So her work, because she was one of the first people to take that on, really ended up being the foundation of works that we continued for quite some time after she left… and so, because of the analysis she did, we were able to make some decisions around continuing to use that data source, which took resources, but also gave us information that we did not have otherwise.” (S6) “So she explored all of this from a surveillance framework, identified many gaps, and made many recommendations for strengthening the system, which has subsequently led to more engagement with the provinces and territories, more engagement with our public health counterparts federally… her work was like a big starting point in terms of helping the team take a look at it, identify shortcomings, and start demonstrating the need to close the gaps and make strong relationships with our public health partners.” (S11)	“We launched a sewage surveillance system of wastewater during COVID that we are trying to expand to other things… So that’s one of the first environmental health file that she took. And with her background as a field epi, when she arrived, I jumped on her, asking if it was something that she thought she could help with because of her background… we were able to dig more into different sewage surveillance system around the world and what type of tools they were using to manage their data… so her background definitely helped.” (M12) “[The graduate] created very impactful ideal spatial mapping that informed where there was increases and clustering of syphilis cases in First Nation communities and also on reserves. This was a gap that our Indigenous partners were looking to have data to support programming and prevention and testing and treatment.” (M8) “Because of this data infrastructure change … [the graduate’s] work strengthening real-time wildfire smoke surveillance made it possible to even have our surveillance running this summer” (M10).
Outbreak investigation and emergency response	During CFEP: 100% (*n* = 22) were involved in supporting or leading field investigations 73% (*n* = 16) strengthened outbreak tools or processes 145 outbreaks were investigated by Trainees (average of 6.6 per Trainee; range 1–50) 100% (*n* = 22) communicable disease outbreaks 59% (*n* = 13) public health emergencies 27% (*n* = 6) non-communicable disease outbreak 5% (*n* = 1) environmental hazards 0% (*n* = 0) natural disasters Following graduation: 65% (*n* = 13) of graduates were involved in supporting or leading field investigations 55% (*n* = 11) communicable disease outbreaks 40% (*n* = 8) public health emergencies 10% (*n* = 2) natural disasters 5% (*n* = 1) environmental hazards Graduates collectively investigated 48 outbreaks (average of one outbreak per graduate every 2 years) 21% (*n* = 4) were mobilized within Canada 5% (*n* = 1) were mobilized internationally 55% (n = 11) agreed that CFEP adequately prepared them for field investigations	“I developed most of the field guides and response documents for COVID-19…in terms of responding to outbreaks, I’ve had a lot of experience.” (G22) “During the COVID pandemic, I was assigned to be the lead epi of one of the community-based responses…the confidence and the experience I got from Field Epi definitely helped me to step up in that role… we developed a clustering [tool] using data science to identify clusters in the community that would be followed up.” (G4) “Some of my work on the disproportionate impact of COVID on First Nation communities in my placement site jurisdiction resulted directly in them being prioritized for vaccine rollout for COVID… the work I did was directly responsible for them being first in line to get the vaccines when they really needed it.” (G10) “I’ve participated in many foodborne outbreaks [which] helped lead to food recalls… by solving the cause of the outbreaks, we protected or prevented further harm to Canadians.” (G2) “The outbreak report that I published has been used as evidence to inform national and international guidance around group A Strep management… that was one of the more direct accomplishments that’s come out of my work as a CFEP field epidemiologist.” (G19)	“He communicated with colleagues...we worked with experts in wind modelling to try to see precisely, from potential sources of Legionnaires’ disease, how it was dispersed in space. It was [the trainee] who made the contacts and worked with the experts on this subject and who produced information for us that was subsequently useful.” (S13) “[She] brought with her experience around outbreaks a lot of structure and actually prompted what we now have implemented as an outbreak response protocol… She brought that structure and made sure that all the outbreak team members knew their roles and responsibilities and the triggers for calling an outbreak… And so she before she left, she did draft for us one of these protocols. And there’s been a lot of work done on it since. But it’s actually an official document we now have to refer to. And that was thanks to [the trainee].” (S5) “She developed a toolkit on outbreak investigations, COVID outbreak investigations, that we posted online for our regional public health partners to use.” (S7) “She was able to be on the ground for some of our outbreak investigation with our Indigenous communities… which really helped in terms of building relationships, but also in terms of gathering data and interpretation, which had an impact in terms of how we rolled out our vaccination campaign for our Indigenous communities… which was extremely valuable and kind of served as a model for other health authorities…she was very robust and very diligent in terms of her documentation… we are still using some of what she generated today.” (S4)	“He definitely was much valued support…and was able to seamlessly participate in the pandemic response…he really increased our capacity during that time… he helped improve our outbreak investigation… he saw through the entire investigation, led it, and worked with our provincial lab partners to utilize some genomic data to understand how COVID was spreading in a food processing plant with over a hundred people… he helped us set up a process on how to support similar workplace clustering events in community during the second wave of the pandemic.” (M2) “She’s passionate [investigating zoonotic outbreaks], which shows in her work … We’ve made quite a lot of progress in this area over the last 2 years. [She] is a key reason why we have been able to do that” (M6).
Data analytics	Following graduation: 95% (*n* = 18) of graduates were involved in data collection and/or analysis 95% (*n* = 18) descriptive analysis 64% (*n* = 15) analytic analysis 79% (*n* = 15) collecting quantitative data 32% (*n* = 6) analyzing qualitative data 26% (*n* = 5) collecting qualitative data 5% (*n* = 1) conducting mixed methods analysis 79% (n = 15) agreed that CFEP adequately prepared them for data analysis 63% (*n* = 12) agreed that CFEP adequately prepared them for data collection	“One of the big parts of my role… was to take their existing data collection tools and improve them. Making some things automated so that they are less prone to error. These are the kinds of things that we learned during field epi and that I was able to put into practice.” (G10) “What has had the biggest impact, and will continue to have impact, is this move to R and leveraging automation for public health. I do not think I would have done that without the field epi program.” (G22) “For our measles response we were able to automate most of our products within a week and a half which meant nobody had to work overtime. It saved staff burnout and made all of our data consistent… Senior management was able to have things in real time… so that was a change that resulted from CFEP.” (G22) “We had a social network analysis training during CFEP, and while social network analysis is not applicable to foodborne illnesses, I managed a way to make the same visuals. So, rather than case-to-case, it’s case-to-exposures. I’ve used that type of tool to improve our data visualization” (G20).	“He basically mapped all of the different facilities and what services they provided and then created a tool so that they could quickly look at a particular service type and see visually on a map where they were offered and where they were not being offered. So if there was a big area that had no place nearby that was providing that service, then that allowed the STI team to be able to make adjustments and considerations for how they might make that service more accessible to individuals in those remote areas.” (S12) “She was really good at taking data to help inform public health action... The data she provided helped inform like our immunization campaign and actually changed some of the scheduling by targeting those populations most in need.” (S5) “All of these initiatives or projects she undertook were priority projects upon which decisions were made by our branch… that information has been used to inform the departmental approach to increasing vaccination amongst [target populations]. Her analysis has informed our understanding of why the vaccine uptake or the documentation of vaccination status… is so low, and then is informing options to proceed with. The analysis that she was doing was filling a gap in terms of really building evidence to inform decisions related to health protection and health promotion.” (S11)	“Rigor, I think it’s definitely something that she brought in in terms of process, like finding way to process data and transforming that into dynamic models that can help us understand situations in a non-emotional way, like in a very neutral way because it’s based on data.” (M12) “She’s able to bring recommendations that are anchored in [data], not invented out of her hat, which are based on the actual regional situation, what is happening in our population right now. So it’s a much more tailored recommendation that represents the reality [and] context that we are working in” (M12). “She played a mentorship role in that piece. So she worked with that junior data analyst and changed the way she was managing the data. She added validation rules… she contributed positively to our workplace culture.” (M11) “With our syndromic emergency department surveillance system, we were translating it from SAS to an R platform. [Graduate] was overseeing that project, it is still being used to this day, regularly by our team members, to monitor respiratory illness in community for us… he now basically is our in-house expert, and he is also trying to incorporate data science approaches to improving our [Legionella surveillance].” (M2)
Non-technical skills	Following graduation: 84% (*n* = 16) of graduates agreed that CFEP adequately prepared them for communicating epidemiological information 63% (*n* = 12) reporting they had communicated epidemiological findings since graduating (95 oral presentations in total) 58% (*n* = 11) had published a paper in a peer-reviewed journal 82% (*n* = 18) reported transferring technical knowledge and skills acquired through CFEP to colleagues at their placement site	“All those soft skills, like how to communicate, how to interact with different public health partners, realizing the need for collaboration, to network, how you clearly communicate an idea in written or verbal form. We did a lot of that in the field epi. So I think those kind of skills that I apply to my day-to-day work quite often… they really help with my day-to-day activity now in my role.” (G2) “We had trainings in various leadership and communication skills. And I’ve found that that’s been really relevant for my work.” (G14) CFEP prepared me for doing oral presentations and writing reports that summarize public health information and make recommendations to senior leadership and other partners. I do this frequently in my current job, with little to no anxiety or stress, and this is due to CFEP training” (G8).	“They had experience in the peer-reviewed publication space, and I think a lot of team members did not have that experience, so they were able to support and help move a lot of things through.” (S15) “She also brought a lot of skill and made lots of contributions just in being a think-on-your-feet kind of person, being able to communicate really well… which is important.” (S11) “[Graduate] was very proactive, quite the go-getter, and keen to get the work done, very organized and methodical and had a great attitude” (S12).	“She’s highly organized, highly flexible. She can pivot quickly. She works well under pressure. It’s all the soft skills more than the technical skills because of the nature of our work.” (M3) “She’s very good at presenting the information in a concise way, providing options as well as recommendations… She engages others to find solutions… She has excelled. I really see her being able to jump in at an issue, address it right away, her resilience. I think it’s all been built as part of the program.” (M7) “She’s one of the best people I’ve ever worked with in my whole life… She’s really strong on the soft skills…She’s really able to just take any task you give her and take it on. It does not matter what it is. She just oozes initiative.” (M6)

#### 3.5.1 Disease surveillance

During CFEP, almost all trainees evaluated, enhanced, or implemented a surveillance system (Table 6). Interview findings demonstrated that the program built strong technical capability in surveillance system design, implementation, and evaluation. As one graduate noted, “before CFEP, I had no experience in surveillance; through CFEP, we had a very in-depth surveillance training, it was very, very great training” (G10). Another emphasized that these skills enabled transition into a surveillance leadership role: “CFEP equipped me with the knowledge to be able to support surveillance and now I am one of the primary epidemiologists conducting routine surveillance here at the Local Health Department (G4).

Graduates reported applying their CFEP skills to support and strengthen a wide range of surveillance systems, including modernizing outdated systems and driving digital transformation. They used their skills to integrate data sources, build dashboards and enhance data visualization. The COVID-19 pandemic provided a unique opportunity for graduates to apply these skills at regional, provincial, national, and international levels. Several graduates described developing new surveillance systems during COVID, including COVID-19 wastewater surveillance systems. In one case, wastewater surveillance was expanded beyond COVID-19 to include other respiratory pathogens, generating data used to “inform recommendations around infection prevention and control messages in the community, as well as to encourage COVID or flu vaccination” (G19).

#### 3.5.2 Outbreak and emergency response

From 2018–2023, all CFEP trainees (100%, *n* = 22) and 65% (*n* = 13) of graduates were involved in supporting or leading field investigations either through PHAC, their placement sites, or at their current organizations (Table 6). Graduates reported that outbreak response opportunities, including mobilizations, were integral for developing skills and building confidence, with one stating that their mobilization “was very helpful in being able to rapidly gather information in an unfamiliar environment, to quickly engage stakeholders, and talk to a variety of organizations” (G15). CFEP’s outbreak investigation training was recognized for strengthening both technical competencies and skills in leadership, coordination and communication.

Trainees were recognized for providing high level expertise during outbreak investigations. According to the placement site supervisors, they applied advanced statistical analyses to identify outbreak sources and inform control measures, such as wind-modelling and geospatial mapping and analysis during a Legionella outbreak. Many assumed leadership roles, improving response timeliness by streamlining processes, redesigning databases and automating analysis and reporting. They also revised outbreak response protocols, developed toolkits and engaged with First Nations communities to build relationships and implement public health control measures.

Graduates were described by managers as being well prepared to lead and support response activities, applying their skills across a wide range of disease outbreaks (Supplementary Material), the COVID-19 response, and other public health emergencies. They introduced improvements in outbreak investigation processes, including improving data accuracy and efficiency through the use of QR codes, developing a natural language processing algorithm to rapidly detect linked COVID-19 cases from free-text interview data, applying modelling tools during a Legionella outbreak, using genomic data to understand transmission dynamics during COVID-19, developing an online toolkit to support respiratory outbreak investigations in industrial settings, and updating response guidelines for COVID-19, measles, influenza, Group A Streptococcus, and zoonoses. Control measures led by graduates included advocating for the prioritization of COVID-19 among disproportionately affected First Nations communities, providing epidemiological evidence to support food recalls and public alerts, and lowering water contamination thresholds to prevent norovirus in shellfish harvesting areas.

#### 3.5.3 Data analytics

During CFEP, trainees applied their data skills to collect data, develop databases, manage large datasets, conduct geospatial and social network analyses, run multivariate regression models, automate reporting processes, and enhance the detection of signals for potential outbreaks. One trainee used geospatial data to inform infection control measures and guide the location of field clinics and distribution of medicine during an emergency response.

Supervisors reported that trainees introduced new analytical tools and approaches, particularly the use of R and advanced data visualization. CFEP trainees transferred their knowledge and skills to workplace colleagues, including providing R training and establishing peer groups to support learning. They applied statistical and geospatial methods to strengthen public health programs, such as improving accessibility to clinics providing sexually transmitted infection services, evaluating the impact of flooding on gastrointestinal illness, analyzing COVID-19 risk factors, and guiding targeted immunization strategies. One trainee applied their geospatial data skills to identify vulnerable populations during extreme weather events.

Following graduation, data and analytical skills continued to be applied widely to routine public health practice. Graduates emphasized the value of programming skills gained through CFEP, particularly proficiency in R. One reflected that, “the most critical skill I learned through CFEP is statistical programming in R… applying R in my day-to-day work is the biggest skill set that I took with me from CFEP, and that I’ve continued on ever since” (G2). Graduates described actively supporting capacity-building within their organizations with one reporting that they trained, “hundreds and hundreds of people in R”, resulting in some health units completely converting to using R (G9). Similarly, another graduate shifted their team to using R and used it to automate most of their data processes. Graduates generated and used epidemiological data and intelligence to inform national vaccine guidelines, update vaccine monographs, target vaccine strategies during pertussis outbreaks, guide tuberculosis elimination strategies and funding decisions, and inform COVID-19 isolation protocols for First Nations communities. Several graduates reported a broader cultural shift within their teams toward evidence-informed decision-making, with epidemiological analysis increasingly viewed as core to effective public health practice.

#### 3.5.4 Non-technical skills

Graduates emphasized the important role of CFEP in strengthening their non-technical and professional skills (Table 6). They reported developing skills and confidence in public speaking and written communication, including preparing epidemiological summaries, public health alerts, peer-reviewed publications, and infographics. CFEP was recognized for enhancing skills in time management, task prioritization and delegation, particularly in high-pressure situations such as outbreak investigations. Graduates reported applying their skills to lead meetings and manage multi-jurisdictional teleconferences during outbreaks. However, a few graduates perceived that the leadership training provided was insufficient to prepare them for supervisory responsibilities assumed after graduation, suggesting a need for more structured leadership development within the program.

Supervisors highlighted the ability of trainees to communicate effectively to a diverse range of audiences and maintain key strategic partnerships. Trainees were recognized for willingness to share their knowledge and skills with colleagues. Current managers similarly appreciated the role of graduates in designing and delivering training on outbreak investigation, emergency management, R, and other technical areas.

#### 3.5.5 Professional outcomes and unexpected benefits or consequences

Overall, 84% (*n* = 16) of graduates indicated that their CFEP training helped them secure their current position. Most (89%, *n* = 17) reported that they continued to use skills gained through CFEP, and, an equal proportion (89%, *n* = 17) agreed that applying CFEP skills benefited the organizations they have worked with. Following CFEP, 37% (*n* = 7) of graduates pursued additional training, including training with WHO Global Outbreak Alert and Response Network (GOARN), undertaking French language training, project management and leadership courses, PhD study, medical training, and supervisory and management development programs.

Graduates reported a range of unexpected outcomes, both positive and negative, arising from their participation in CFEP. Many commented on personal confidence, professional credibility and enduring networks that promoted professional and personal support. One graduate stated that CFEP, “gave me a lot of the skills and confidence that I needed to come back to my team in a higher position. CFEP really prepared me to have the leadership skills and the confidence that I needed” (G10). Others highlighted the value of CFEP’s reputation in enabling career advancement: “The reputation of the strength of the program helped me get this position, that was an unexpected impact of the training” (G16). Several described mobilizations and professional connections that opened international opportunities, including WHO roles, with one noting that their CFEP mobilization experiences, “100 percent opened up the door to work with WHO on their COVID-19 surveillance” (G6). Graduates also reflected on the profound personal and cultural impacts of their mobilization experiences: “From a personal perspective, I think having the experience of the deployments really sort of helps your personal maturity. It’s not just a work experience, you’re also going somewhere, living there, seeing a new culture… it opens your mind and that has had a profound effect on me” (G4). The extent to which CFEP influenced career direction and advancement was another unexpected outcome: “It was a life-changing experience to do the field epi program, and I have no regrets, only gratitude. It changed the trajectory of my career and has had impacts on my life as well” (G4). Another added: “I’m very grateful, [CFEP] has totally changed my career” (G13).

However, graduates also reported several negative impacts, primarily relating to mental health. Graduates reported heavy workloads, isolation, burnout, and limited support during the COVID-19 pandemic. Some graduates reported experiencing persistent mental health challenges and burnout following graduation. One stated: “We had such a hard time living through field epi during COVID, some had to take mental health leave. Like, it was very severe… and very unique to the time that we served” (G10). Despite the challenges of the pandemic, this graduate also noted that the overall CFEP experience was “overwhelmingly positive” (G10).

The program’s requirement for mobility, including to the placement site and for short-notice mobilizations, was described as disruptive, affecting relationships and stability. One explained: “During the two years that you’re in the training program, it’s so intensive that you don’t really have the chance to build roots anywhere because you are travelling so much. You’re traveling for training, you’re traveling for mobilizations, you’re traveling for outbreak investigations. So it was, I guess, tough on my social life” (G6). Some also reported challenges with placement sites, including unsupportive supervision, unmet expectations, and missed opportunities to apply their field epidemiology skills, which they found disappointing and unexpected.

### 3.6 Alumni network

All graduates (*n* = 24, 100%) reported interacting with other graduates as part of an informal alumni network: 25% (*n* = 6) very frequently, 42% (*n* = 10) frequently, 25% (*n* = 6) occasionally, and 8% (*n* = 2) rarely. Most used the networks for personal support and advice (72%, *n* = 13) and for serving as a sounding board to validate ideas or troubleshoot problems (67%, *n* = 12). Half of the graduates (*n* = 9) found the network useful for sharing professional development or other training opportunities, and 44% (*n* = 8) for sharing technical knowledge and skills.

### 3.7 Observations and recommendations

#### 3.7.1 Graduates

A summary of the key recommendations made by graduates, supervisors, and managers is shown in Table 7. These included ongoing access to training material and the development of an online repository of past training content. Graduates also advocated for the establishment of a more structured and formal alumni network, with three-quarters (75%, *n* = 18) indicating that such a network would be valuable. Graduates envisioned a formal alumni network as a key mechanism for sharing job and professional development opportunities, maintaining up to date contact details, sharing technical resources, organizing webinars and workshops, and hosting in-person gatherings. Several highlighted the importance of maintaining and strengthening field epidemiology knowledge and skills post-graduation and recommended the provision of ongoing courses and workshops. Other recommendations included creating a roster for domestic and international mobilizations, strengthening connections between alumni and agencies requiring field epidemiology expertise, involving more graduates as CFEP mentors and trainers, and providing additional support for career development.

**Table 7:** Key recommendations made by CFEP graduates, placement site supervisors and current managers, 2024.

Particpant group	Key recommendations
Graduates	Recognize prior experience when determining initial salary for trainees Establish a more transparent CFEP recruitment process Expand leadership training during CFEP Include or expand training on advanced analytics, R, GIS, infectious diseases, data collection, qualitative methods Provide an option for trainees to personalize training options / tailor learning to individual needs Ensure face-to-face learning remains a core part of CFEP Expand the variety of placement sites for trainees Strengthen assessment of placement site capacity and supervision quality Provide placement site supervisors clearer project outlines Offer training and mentorship support for placement site supervisors Provide more transparent and equitable access to mobilization opportunities Expand national and international mobilization opportunities Enforce post-mobilization rest periods Create more opportunities for formal and informal networking during and after CFEP Establish a more formal/structured alumni network Provide post-graduation engagement opportunities, such as events, webinars, refresher training, and mobilizations (create a roster for domestic and international mobilizations) Provide post-graduation career transition support, including guidance on federal hiring processes Provide post-graduation support for publishing program related work Create an alumni portal with a repository of resources Increase engagement of alumni as program mentors and trainers Extend CFEP program by offering opportunities to develop higher levels skills that are not currently part of CFEP
Supervisors	Streamline application process Strengthen training in non-technical skills (communication, resilience) Expand French speaking candidate availability Strengthen rapid mobilization mechanisms
Managers	Provide more frequent mobilization opportunities Establish a formal CFEP network Expand graduate employment opportunities beyond the federal level

#### 3.7.2 Supervisors

Supervisors spoke highly of CFEP and consistently emphasized the value that trainees brought to placement sites, with one stating: “We’ve been very happy with the placements that we’ve had over the last 7 or 8 years. All of them have been exceptional, they’ve brought in new ways of working. We’ve been happy with the program overall” (S1). Supervisors highlighted the reciprocal value of CFEP placements, with trainees gaining applied field experience and sites benefiting from knowledge transfer, process improvements, and enhanced outbreak response capacity. Supervisors expressed strong support for the ongoing future of the program, recognizing its national importance: “It’s such an important part of the public health landscape in Canada. I just wish there was more support for the program and that more trainees could be placed every year” (S9).

Some supervisors observed a shift in the background of incoming trainees, with more coming from research backgrounds, highlighting the importance of ensuring that the training and trainee selection processes remain aligned with the practice-based principles of applied field epidemiology.

Some supervisors noted that the CFEP administrative processes for hosting trainees were burdensome and could deter new placement sites from hosting trainees. They recommended simplifying and streamlining the administrative requirements to enable greater participation. Other recommendations included strengthening non-technical skills training to support trainees’ communication, resilience, and adaptability to fast-paced environments. Additional suggestions included expanding the pool of French-speaking candidates, establishing a formal alumni network, and strengthening mobilization mechanisms for outbreaks and public health emergencies.

#### 3.7.3 Current managers

Current managers described graduates as being well prepared for roles in public health and having strong analytical and critical thinking skills. One manager stated: “We’ve had a number of CFEP trainees come through during my time, and they’ve all been pretty excellent… the program does a great job of recruiting good trainees” (M10). Another noted the positive influence graduates have on workplace culture and performance, “raising the bar of work and expectation from those around them… elevating the whole team’s skills because they are also learning alongside the field epi” (M8). Graduates were viewed as preferred hires due to their specialized training and practical experience, and adaptability to complex public health environments.

Managers recommended that graduates be provided with more frequent mobilization opportunities following CFEP, recognizing that field experience is critical for maintaining capability and confidence over time. Managers also identified opportunities to strengthen written and verbal communication, particularly in tailoring messages for diverse audiences and developing succinct briefing notes for senior management. Like placement site supervisors, managers highlighted the need to build the resilience, confidence and assertiveness of graduates to ensure they can operate confidently in high-pressure situations alongside senior clinicians. Expanding graduate employment opportunities beyond the federal level was also suggested to maximize the program’s contribution to national field epidemiology capacity.

## 4 Discussion

Our findings show that CFEP has made an important contribution to strengthening Canada’s public health capacity. Guided by the program theory of change, this evaluation examined the outputs, outcomes and impacts of CFEP using contribution analysis. Rather than attributing effects directly to the program, we assessed whether CFEP plausibly contributed to observed changes by examining alignment between expected and observed outcomes, temporal sequencing, and consistency across data sources. Survey and interview data were triangulated and alternative explanations considered. The program theory proposes that high-quality, work-integrated field epidemiology training and mentoring build confidence and competence, leading to routine application of skills, strengthened field epidemiology functions, and, over time, strengthened health-systems contributing to improved health outcomes.

Graduates, placement site supervisors, and current managers all reported substantial improvements in technical competencies and the application of skills to improve public health. Graduates transferred knowledge and skills in their placement sites and workplaces, contributing to a culture of evidence-based decision making. Most continued to be employed in public health roles, with many moving into supervisory and mid-level management positions. These high retention rates, which are consistent with observations made by other FETPs (27–29), contribute to the cumulative outcomes and impacts of the program.

Graduates reported having a high level of confidence in performing a range of field epidemiology activities across CFEP’s professional experience guidelines. Graduates, placement site supervisors and current managers provided examples of how skills were used in response to emerging public health issues. Our findings align with other evaluations documenting the important role of FETPs in increasing the knowledge and skills of trainees (19, 20, 23–25, 27, 30–32) to drive and support evidence-based decision making (19, 24, 25, 27, 30), improve surveillance (20, 23–25, 30, 31), strengthen outbreak response (20, 25, 26, 29, 30, 33), and enhance capacity to respond to public health emergencies and disasters (20, 33). Consistent with the findings of others, the training also contributed to the development of non-technical skills, including communication and leadership skills (24), and facilitated the formation of strong professional networks and partnerships (24, 25, 27, 29). Areas where graduates expressed less confidence included managing large outbreak investigations and conducting analytic studies. Providing additional outbreak response opportunities during and after training would help build confidence and competence in these areas. Strengthening partnership with international agencies, such as WHO GOARN, Médecins Sans Frontières or the Canadian Red Cross, could be considered as one mechanism for expanding the number and variety of mobilization opportunities. Refresher training following graduation, as recommended by graduates, would provide additional opportunities to reinforce and further develop key field epidemiology competencies.

Trainees and graduates actively supported a range of public health functions across a range of placement sites and workplaces. CFEP’s inclusion of placement sites in underserved, rural and remote regions promoted equitable contributions to health system strengthening. Trainees and graduates evaluated and improved a wide range of disease surveillance systems and led numerous outbreak investigations and emergency response activities across Canada and internationally. The high proportion of CFEP trainees and graduates deployed to support federal, provincial, and territorial partners demonstrates the central role of CFEP in developing the surge capacity to meet these demands. The improved capacity to respond, and the large number of outbreaks investigated by FETP trainees and graduates, has been previously highlighted (3, 20, 25, 26, 29, 30, 33, 34). The wide range of outbreaks investigated by trainees has also been described by others (3, 4, 34–46). The important contribution of CFEP trainees and graduates to the COVID-19 response reflect findings from other programs (47–51). Hu et al. (2022), for example, surveyed 64 FETPs and found that trainees provided critical assistance across a range of areas during COVID-19, including conducting epidemiological studies, managing logistics and coordination response activities, leading risk communication efforts, and delivering training (47).

Graduates were noted for improving processes, developing guidelines, automating systems and introducing a range of innovations, many of which were adopted permanently, leading to long-term program improvements. These findings support those of Dey et al. (2019), where UK FETP graduates were recognized for introducing innovations and undertaking work that led to changes in practice, particularly in the use of statistical tools and techniques, and the automation of reporting (27). Dey et al. (2019) highlighted how the UK FETP strengthened health systems directly through strengthening the national public health workforce, and indirectly by influencing behaviors and skills across the broader health workforce (27). The sharing of knowledge and skills was an important finding of this evaluation, and supports outcomes described by Dey other FETP evaluators (25, 27, 31).

The strong networks developed through CFEP were identified by several graduates as a key unintended positive consequence of participating in CFEP. These networks served as important conduits for sharing information, providing expertise, and troubleshooting problems. In-person interactions were considered essential for developing these peer-to-peer networks. The value of the professional networks developed through field epidemiology training has been previously reported. Dick et al. (2014) found that 85% of alumni and 86% of mentors in the U.S. maintained relationships beyond the duration of the training program. In Europe, a formal FETP alumni network was noted for facilitating the exchange of information and strengthening collaborations during major health emergencies such as Ebola, COVID-19, and mpox (52). Developing a more formal alumni network was one of the recommendations made by graduates, placement site supervisors and current managers. Whilst many national FETPs maintain informal networks, there are also a few formal ones, the oldest of which is the United States Epidemic Intelligence Service Alumni Association. This network manages alumni activities, shares job opportunities, provides career mentoring, hosts leadership events, and supports several EIS awards (53). The European Program for Intervention Epidemiology Training (EPIET) Alumni Network is another formal network offering technical support and ongoing mentoring to trainees and alumni from across Europe. It also contributes to the design, delivery and promotion of training, and supports a number of events, including the European Scientific Conference on Applied Infectious Disease Epidemiology (54). The Field Epidemiology Training Program Alumni Foundation, Inc. (FETPAFI) is a non-profit organization providing epidemiologic services in research, training, and disease surveillance in the Philippines (55). These formal networks require considerable coordination and logistical support and are managed separately from FETPs.

The impact of the pandemic response on trainees’ mental health emerged as an important unexpected negative consequence faced by trainees. The impact of the pandemic on other frontline responders and health workers has been well documented, with high rates of post-traumatic stress disorder, depression, anxiety and insomnia linked to heavy workloads, moral distress and demoralization (56–58). The review by Hooper et al. (2021) emphasized that emergency response workers often experience delayed and cumulative psychological harm, and that structured, proactive interventions were more effective than *ad-hoc* self-care resources (57). Incorporating mental health preparedness and psychological first aid training modules may mitigate the long-term mental health effects experienced by trainees during future outbreak or public health emergency responses. CFEP could also formalize strategies recommended by graduates and managers, including enforcement of post-mobilization rest periods and training to strengthen the resilience of graduates when operating in high-pressure situations.

Considering the large number of FETPs globally, and the importance of these programs in developing national public health workforces, there are relatively few published evaluations focusing on outcomes and impacts (59). Evaluations provide evidence of a program’s effectiveness and relevance, guiding program improvement and providing accountability to donors and partners. They also generate evidence that can be used to advocate for the program and raise awareness of the valuable contributions of trainees and graduates. Several FETP evaluations have linked the underutilization of graduates to poor recognition of the value they bring to the workplace (19, 20). As FETPs aim to improve population health by strengthening the capacity to detect, investigate, and respond to public health threats, understanding how FETPs contribute to these outcomes is essential. Theory-based evaluation approaches are well suited to assessing the outcomes and impacts of FETPs (60–62). Rather than attempting to quantify impact by comparison to a counterfactual (non-intervention control group), theory-based approaches collect evidence to build a credible narrative of the program’s contribution to observed outcomes and impacts along the program’s change pathway. Contribution analysis, while unable to prove causality, reduces uncertainty around the extent to which a program has contributed to changes that have occurred (63, 64).

The results documented by this evaluation aligned with what was expected based on the program theory (14). There were, however, other factors which may have influenced and contributed to the outputs and outcomes described. The unprecedented demand for epidemiological expertise during the COVID-19 response provided trainees and graduates with extensive opportunities to apply and expand their skills, accelerating outcomes that may not have occurred under regular conditions. Increased investments in disease surveillance and emergency response, especially during COVID-19, may have also contributed to the outcomes described by graduates. Non-CFEP training within placement sites and workplaces, as well as further professional development opportunities pursued by some graduates, may have reinforced or expanded the skills of graduates contributing to their achievements. General advances in technology and shifts in public health priorities may have also contributed to the results attributed to CFEP. Despite these factors, the information from surveys and interviews highlight the primary role of CFEP in contributing to the learning, behaviors, and results described by graduates. While measuring morbidity or mortality change was beyond the scope of this evaluation, early indicators demonstrate plausible progress along the impact pathway. In many instances, graduates, placement site supervisors, and current managers described a direct link between the skills acquired through CFEP, the application of these skills in the workplace, and the subsequent results.

This study has several limitations. We acknowledge the potential for non-response bias. Graduates not participating may have been less engaged with CFEP or less supportive of the program, leading to an overestimation of positive outcomes. The evaluation also relied on self-reported behaviors and impacts without independent verification. Responses may be subject to recall and social desirability bias, potentially leading some graduates to overstate their contributions or successes. The potential for social desirability bias was reduced by using an independent interviewer with no program affiliation and clearly communicating the process for maintaining confidentiality. Interview guides were also designed to elicit both positive and critical reflections. The inclusion of graduates from multiple training years offered a broad perspective but also introduced variability in the timeframe available for graduates to apply skills and demonstrate impact. The study’s cross-sectional design further limits conclusions regarding the long-term sustainability of outcomes. While triangulation of quantitative and qualitative data strengthened the credibility of findings and mitigated some limitations inherent in single-method approaches, these constraints should be considered when interpreting results (65). Reflexivity was an important component of this evaluation. The research team’s professional backgrounds and experiences may have shaped the interpretation of findings. To address this, multiple strategies were used to enhance credibility, including ongoing critical reflection, discussion of interpretations among team members with diverse disciplinary perspectives (epidemiology, qualitative social science, and program evaluation), and deliberate attention to both positive and negative accounts by participants. Interview questions were also designed to minimize leading responses. Although the research team’s familiarity with the field may have supported nuanced interpretation, it may also have introduced assumptions about program value. Transparency in analytic decisions and comparison of quantitative and qualitative data were used to mitigate these influences.

It is recommended that future research focus on the long-term sustainability of outcomes and impacts. Longitudinal studies tracking a selection of graduates would provide insights into career progression, retention and influence within the public health service, and the sustained application of field epidemiology skills. Given the mental health challenges reported during large scale response activities, future studies could explore and assess the effectiveness of interventions and support mechanisms to enhance resilience, wellbeing and post-mobilization support. Finally, comparative studies between FETPs in different countries could help identify program design features that are most strongly associated with sustained outcomes and impacts. Developing a core set of evaluation indicators would facilitate inter-program comparisons.

The contributions of applied epidemiology to contemporary public health challenges, such as vaccine hesitancy and uptake (66, 67) or health-system pressures associated with post-COVID syndrome (68), reinforce the value of FETPs as a strategic investment rather than solely a technical training activity. This study has shown how CFEP has strengthened Canada’s public health workforce to address public health priorities by equipping health professionals with field epidemiology competencies. Graduates contributed to supporting and enhancing disease surveillance, outbreak response, and program delivery. This evaluation provides evidence to indicate that CFEP has met its objectives of developing an agile workforce capable of responding to emerging public health issues and improving health systems in Canada. Additional response opportunities and resilience training would further enhance CFEP’s role in developing Canada’s field epidemiology workforce.

## Data Availability

The raw data supporting the conclusions of this article will be made available by the authors, without undue reservation.
