# Supplementary material for: Strengthening field epidemiology capacity in Canada: a mixed-methods evaluation of the Canadian field epidemiology program
Source: Front Public Health. 2026 Mar 20;14:1777134. doi: 10.3389/fpubh.2026.1777134 (PMC13047194; doi:10.3389/fpubh.2026.1777134)
Supplement: Supplementary file 1 [file Data_Sheet_1.pdf]

Evaluating the Impact of the Canadian Field Epidemiology Training Program

Graduate Survey

V4.0

INTRODUCTION

The Public Health Agency of Canada and the University of Newcastle are conducting an evaluation of the Canadian Field Epidemiology Program (CFEP). As a graduate, we are interested in your experiences during and following your training. The information you provide will be used to inform the development of Canada's field epidemiology training program.

Please refer to the Participant Information Sheet [add link] for further information about this survey.

CONSENT

I have read the information sheet linked above.

I understand that the survey will be conducted as described in the Participant Information Statement, a copy of which I have read and understood.

I understand that I can withdraw from the evaluation as explained in the Participant Information Statement, and I do not have to give any reason for my decision to withdraw.

I understand that my personal information will remain confidential to the researchers. I have had the opportunity to have questions answered to my satisfaction.

I agree to participate in the above research project and give my consent freely.

- ☐ Yes
- ☐ No

INTRODUCTION

1. What year did you graduate from CFEP?
   [background]
   - 2018
   - 2019
   - 2020
   - 2021
   - 2022
   - 2023
   - Other, please specify: ______

2. What stream did you graduate from?
   [background]
   - EC
   - MD
   - VM
   - Other, please specify: ______

3. Where were you placed during CFEP (the primary placement site where you spent the majority of your time during CFEP)?

[background]

____

4. How many years did you work in a public health-related position before starting CFEP? [background]

   - _______ years
   - I didn't work in public health prior to CFEP
   - Don't know

5. How many years have you worked in a public health-related position since graduating from CFEP? [background]

   - _______ years
   - I haven't worked in public health since graduating from CFEP
   - Don't know

   [if haven't worked in PH] Have you worked since graduating from CFEP?

   - Yes
   - No [skip to Q8]

   [if yes] What field(s) of work have you worked in? ________________________

   [if yes] How useful has the knowledge and skills gained from CFEP been for the work you have since since graduating?

   - Not useful
   - Slightly useful
   - Moderately useful
   - Very useful
   - Extremely useful

6. Are you currently employed?

   - Yes
   - No

   [If yes] What is the name of your current employer? ___________________

   [If yes] What is your current position title? _______________________

   [If yes] Which of the following position roles best describes your current management responsibilities? (check all that apply)

   - Non-managerial
   - Program manager
   - First-line manager (manages technical staff)
   - Middle level manager (manages staff who manage other individuals)
   - Top level manager / executive
   - Other, specify:_________________________

7. Whilst working in public health, what types of employers have you worked for (including your current employer if relevant)? (check all that apply)

[Indicator 5.1]

- ☐ Government – Federal
- ☐ Government – Provincial
- ☐ Government – Local
- ☐ University
- ☐ Non-government organisation (NGO)
- ☐ International organization
- ☐ Private
- ☐ Other, specify: ________________

8. How often do you communicate or interact with other CFEP graduates as part of an informal alumni network?

    - Never
    - Rarely
    - Occasionally
    - Frequency
    - Very frequently

    [If more than never] How useful has this informal alumni network been for:

    Being a sounding board to validate ideas or troubleshoot problems?
    • Useful • Neutral • Not useful • Don't know

    Sharing technical knowledge or skills?
    • Useful • Neutral • Not useful • Don't know

    Receiving and/or giving personal support and advice?
    • Useful • Neutral • Not useful • Don't know

    Sharing professional development or other training opportunities?
    • Useful • Neutral • Not useful • Don't know

    Sharing job openings or opportunities for career advancement?
    • Useful • Neutral • Not useful • Don't know

9. Would having a more formal alumni network be of value to you?

    - Yes
    - No

    [If yes] What should a formal alumni network do?
    [check all that apply]

    - Provide contact details of CFEP graduates
    - Provide regular webinars on field epidemiology related topics
    - Provide a platform for sharing technical resources
    - Share professional developing opportunities
    - Share job openings or opportunities for career advancement
    - Other, please specify:

10. Are you currently a member of a public health committee, working group or professional network at the:
    [Indicator 5.5]

Local level	• Yes • No
Provincial / Regional level	• Yes • No

National level • Yes • No
International level • Yes • No

[If yes to any of the above] Please specify the name(s) of the committee, working, group or network:

____

LEARNING

11. During the CFEP training, how would you rate:
[Background]

	Very good	Good	Acceptable	Poor	Very poor
a. The delivery of the training					
b. The training materials provided					
c. The training content					
d. The knowledge of the facilitators					
e. Support / mentoring during workshops					
f. Supervision by the placement site					
g. Support / mentoring by CFEP during the placement					

12. Can you provide 3 suggestions on how CFEP could be strengthened for future training?
[background]

a. ________________________________

b. ________________________________

c. ________________________________

13. During CFEP, were you involved in supporting an epidemiological investigation, such as an outbreak, public health emergency or disaster response?
[Indicators 1.1, 2.4]

- Yes
- No
- Don't know

[If yes] How many of these investigations did you support? ________

[If yes] What type(s) of investigation did you support during CFEP?
(check all that apply)

- Communicable disease outbreak
- Non-communicable disease outbreak
- Public health emergency

- Environmental hazard
- Natural disaster
- Human-induced disaster
- Other, please specify: ________________

[If yes to public health emergency] What public health emergency(ies) did you respond to?

____

[If yes to environmental hazard] What environmental hazard(s) did you respond to?

____

[If yes to natural or human-induced disaster] What disaster(s) did you respond to?

____

14. During CFEP, were you involved in strengthening outbreak response tools or processes?
[Indicators 1.1, 1.8]

    - Yes
    - No
    - Don't know

15. During CFEP, did you evaluate, enhance, or implement a surveillance system?
[Indicators 1.1, 1.7]

    - Yes
    - No
    - Don't know

    [If yes] Were any recommendations you made implemented?

        - Yes
        - No
        - Don't know

16. During CFEP, were there any unexpected positive outcomes related to your experience in the program, either professionally or personally?
[Indicators 4.1, 4.2]

    - Yes, please specify: ________________________________
    - No
    - Don't know

17. Since graduating from CFEP, have there been any unexpected positive outcomes related to your experience in the program, either professionally or personally?
[Indicators 4.1, 4.2]

    - Yes, please specify: ________________________________
    - No
    - Don't know

18. During CFEP, were there any unexpected negative outcomes related to your experience in the program, either professionally or personally?

[Indicators 4.1, 4.2]

- Yes, please specify: ___________________________________
- No
- Don't know

19. Since graduating from CFEP, have there been any unexpected negative outcomes related to your experience in the program, either professionally or personally?
[Indicators 4.1, 4.2]

    - Yes, please specify: ___________________________________
    - No
    - Don't know

20. During CFEP, were you able to transfer your knowledge and skills to others at your placement site? This could be through, for example, providing training, mentoring or sharing new techniques, tools or approaches with others.
[Indicator 1.2]

    - Yes
    - No
    - Don't know

BEHAVIOUR (KNOWLEDGE AND SKILL APPLICATION)

21. *Please indicate your level of agreement or disagreement with the following statement:*
During my CFEP training, I was able to apply my knowledge and skills in the following domains:
[Indicators 1.1, 2.3]

	Strongly agree	Agree	Neither agree nor disagree	Disagree	Strongly disagree	Don't know N/A
Field investigation						
Epidemiologic analysis						
Public health surveillance						
Peer review publication						
Scientific communication to a variety of audiences						
Oral presentations						

22. During your CFEP training:
[Indicator 1.1]

    a. What key enabling factors supported you apply your knowledge and skills?

    ____

    b. What key barriers that hindered you from applying your knowledge and skills?

    ____

23. Today, how confident would you be conducting the following activities?
[Indicators 3.3, 3.4, 3.5]

Learning outcome	Very confident (I could do this without any support)	Confident (I would need some support)	Not confident (I would need a lot of support)	Don't know
a. Using surveillance data to guide public health programming				
b. Evaluating a surveillance system and making recommendations for system improvement				
c. Leading a small scale (single jurisdiction) outbreak investigation				
d. Leading a large scale (multi-jurisdictional) outbreak investigation				
e. Being deployed into the field to support an outbreak response				
f. Supporting a public health emergency response within an Incident Management System (IMS)				
g. Implementing an analytic epidemiologic study (e.g. cohort or case control study)				
h. Conducting descriptive data analysis				
i. Conducting analytic data analysis				
j. Developing evidence-based policy recommendations from data and information				
k. Giving an oral scientific presentation				
l. Writing an abstract for a scientific conference				
m. Writing a scientific manuscript for a peer-review journal				
n. Creating communication products for a non-scientific audience				

RESULTS

Field Investigation

24. Since graduating from CFEP, have you been involved in supporting or leading an epidemiological field investigation, such as an outbreak, public health emergency or disaster response?
    [Indicators 2.4, 2.5]

    - Yes
    - No
    - Don't know

    [If yes] What type(s) of investigations have you supported or led?
    (check all that apply)

    - Communicable disease outbreak
    - Non-communicable disease outbreak
    - Public health emergency
    - Environmental hazard
    - Natural disaster
    - Human-induced disaster
    - Other, please specify: _________________

    [If yes for communicable or non-communicable disease outbreak] How many disease outbreaks have you investigated? ______________

    [If >0] Of these disease outbreaks, how many

Were you the lead investigator	
Were you a support investigator	
Were you mobilized within Canada (outside your regular place of work)	
Were you mobilized outside of Canada	

[If yes to public health emergency] What public health emergency(ies) did you respond to?

____

[If yes to environmental hazard] What environmental hazard(s) did you respond to?

____

[If yes to natural or human-induced disaster] What disaster(s) did you respond to?

____

25. In your current role, how often are you involved in field investigations
    [Indicator 2.5]

    - Very Often
    - Often
    - Occasionally
    - Rarely
    - Never

26. *Please indicate your level of agreement or disagreement with the following statement:* CFEP adequately prepared me for conducting field investigations
[Background]

- Strongly agree
- Agree
- Neither agree nor disagree
- Disagree
- Strongly disagree
- Don’t know / not applicable

27. Please provide any examples of how your practice in this area has changed as a result of CFEP
[Indicator 1.8]

____

Surveillance

28. Since graduating from CFEP, which of the following disease surveillance activities have you been involved in? (check all that apply)
[Indicators 1.7, 1.9]

☐ Managing a surveillance system
☐ Analysing data from a surveillance system
☐ Using surveillance data to detect outbreaks
☐ Using surveillance data to inform public health programming
☐ Using surveillance data to develop new or modify existing health policy
☐ Evaluating a surveillance system
☐ Improving a surveillance system,
☐ Implementing a new surveillance system
☐ Mass gathering surveillance
☐ I have not been involved in disease surveillance

29. In your current role, how often are you involved in disease surveillance activities
[Indicator 1.9]

- Very Often
- Often
- Occasionally
- Rarely
- Never

30. *Please indicate your level of agreement or disagreement with the following statement:* CFEP adequately prepared me for supporting disease surveillance activities
[Background]

- Strongly agree
- Agree
- Neither agree nor disagree
- Disagree
- Strongly disagree
- Don’t know / not applicable

31. Please provide any examples of how your practice in this area has changed as a result of CFEP
[Indicator 1.9]

____

Data collection and analysis

32. Since graduating from CFEP, which of the following data collection and analysis activities have you been involved in? (check all that apply)
    [Indicator 1.6]

    - ☐ Collecting quantitative data
    - ☐ Collecting qualitative data
    - ☐ Managing small databases (<100 records)
    - ☐ Managing medium databases (100 - 1000 records)
    - ☐ Managing medium databases (1000+ records)
    - ☐ Analysing quantitative data - descriptive
    - ☐ Analysing quantitative data - analytic
    - ☐ Analysing qualitative data
    - ☐ Conducting mixed-methods analysis

33. In your current role, how often are you involved in data collection
    [Indicator 1.6]

    - Very Often
    - Often
    - Occasionally
    - Rarely
    - Never

34. In your current role, how often are you involved in data analysis
    [Indicator 1.6]

    - Very Often
    - Often
    - Occasionally
    - Rarely
    - Never

35. *Please indicate your level of agreement or disagreement with the following statement:*
    CFEP adequately prepared me for <u>data collection</u>
    [Background]

    - Strongly agree
    - Agree
    - Neither agree nor disagree
    - Disagree
    - Strongly disagree
    - Don’t know / not applicable

36. Please provide any examples of how your practice in this area has changed as a result of CFEP
    [Indicator 1.6]

    ____

37. *Please indicate your level of agreement or disagreement with the following statement:*
CFEP adequately prepared me for data analysis
[Background]

- Strongly agree
- Agree
- Neither agree nor disagree
- Disagree
- Strongly disagree
- Don't know / not applicable

38. Please provide any examples of how your practice in this area has changed as a result of CFEP
[Indicator 1.6]

____

Communications

39. Since graduating from CFEP, approximately how many times have you communicated epidemiologic findings through
[Indicators 6.3, 6.5. 6.7]

Peer-review publications (lead author)	
Peer-review publications (co-author)	
Oral presentations (conference)	
Oral presentations (other)	

40. In your current role, how often are you involved in communicating epidemiological information (through reports, papers presentations etc)
[Indicators 6.3, 6.5. 6.7]

- Very Often
- Often
- Occasionally
- Rarely
- Never

41. Please indicate your level of agreement or disagreement with the following statement: CFEP adequately prepared me for communicating epidemiologic information
[Background]

- Strongly agree
- Agree
- Neither agree nor disagree
- Disagree
- Strongly disagree
- Don't know / not applicable

42. Please provide any examples of how your practice in this area has changed as a result of CFEP

____

OUTCOMES & IMPACTS

43. *Please indicate your level of agreement or disagreement with the following statement:*
During my CFEP training I was able to apply my knowledge and skills in the following domains:

	Strongly agree	Agree	Neither agree nor disagree	Disagree	Strongly disagree	Don't know N/A
CFEP helped me get to my current role						
Taking part in CFEP has helped me achieve my career aspirations						
I am still using skills that CFEP taught me						
Taking part in CFEP has been beneficial to the organizations that I have worked for						
CFEP build my confidence to work in public health / field epidemiology						

44. Please give any examples of how CFEP have helped you, or not, achieve your current role and or career aspirations

____

45. Please give any examples of any other initiatives or training that you feel helped you achieve your career aspirations

____

46. Please give examples of how CFEP has been beneficial to the organization(s) you have work for

____

47. After graduating from CFEP, what areas have you had influence on in your workplace
(check all that apply)
[Indicators 6.8]

- ☐ Detection and response to health threats
- ☐ Disease surveillance
- ☐ Data management
- ☐ Access to health services
- ☐ Service delivery
- ☐ Key performance indicators
- ☐ Standard setting for technical work
- ☐ Communicating epidemiologic findings
- ☐ Providing the scientific basis for health related program and policy decisions
- ☐ Developing health related programs
- ☐ Develop health related policy
- ☐ Other, please specify: __________________________

- ☐ I feel I have not been able to have influence in my workplace

48. Can you give an example of where your work, during or after graduation, has resulted in a change in routine workplace practice?

    - Yes
    - No
    - Don’t know

    If yes, please describe: ____

MOST SIGNIFICANT CHANGE

49. Please think of one of the most important outcomes or changes you have achieved or implemented since graduating which can, in full or in part, be attributed to CFEP:

    a. Describe the outcome, when and where it occurred, and who was involved

    ____

    b. Why is the outcome significant?

    ____

    c. What was your contribution to the outcome’ what was the role other players?

    ____

DEMOGRAPHICS

50. What is your gender?
    [Indicator 6.1]

    - ☐ Male
    - ☐ Female
    - ☐ Non-binary
    - ☐ None of the above, I identify as: _____________
    - ☐ Prefer not to say

51. What age group do you fall into?
    [Background]

    - ☐ <20 years
    - ☐ 20 – 29 years
    - ☐ 30 – 39 years
    - ☐ 40 – 49 years
    - ☐ 50 – 59 years
    - ☐ 60+ years

52. What is your first language?

- English
- French
- Other, please specify:

53. Do you identify as a person with a disability or chronic condition?

- Yes
- No
- Prefer not to answer

54. In our society, people are often described by their race or racial background. These are not based in science, but our race may influence the way we are treated by individuals and institutions. Which category(ies) best describes you? Check all that apply:
[Indicator 6.1]

- Black (African, African Canadian, Afro-Caribbean descent)
- East Asian (Chinese, Japanese, Korean, Taiwanese descent)
- Indigenous (First Nations, Inuk/Inuit, Métis)
- Latin American (Hispanic or Latin American descent)
- Middle Eastern (Arab, Persian, West Asian descent)
- South Asian (South Asian descent e.g., Bangladeshi, Indian, Indo-Caribbean, Pakistani, Sri Lankan) Southeast Asian (Cambodian, Filipino, Indonesian, Thai, Vietnamese, or other Southeast Asian descent) White (European descent)
- Do not know
- Prefer not to answer

Thank you so much for taking the time to complete this survey.

Alignment of survey questions with specific indicators

Indicator Number	Indictor	Survey Question Included
1.1	[Fellows] CFEP Field Epidemiologists were able to apply their field epidemiology knowledge and skills to benefit their placement site [explore enablers and barriers]	x
1.2	[Fellows] CFEP Field Epidemiologists were able to transfer their knowledge and skills to colleagues at their placement site [explore enablers and barriers]	x
1.4	[Fellows] Number/percentage of surveillance system evaluations where one or more of the recommendations made by the Field Epidemiologist have been implemented with one year of the evaluation [explore enablers and barriers; explore significance of change(s); explore fellows' contribution]	x
1.6	[Health system] Graduates contribute to the improvement of data collection, data management, or data analysis tools and processes. [explore enablers and barriers; explore significance of change(s); explore fellows' contribution]	x
1.7	[Health System] Number of disease surveillance systems strengthened by fellows and graduates [explore examples/categories, explore enablers and barriers; explore significance of change(s); explore fellows' contribution]	x
1.8	[Health System] Number of outbreak response tools and practices strengthened by fellows and graduates [explore examples/categories, explore enablers and barriers; explore significance of change(s); explore fellows' contribution]	x
1.9	[Health System] Graduates contribute to strong disease surveillance systems that guide public health programming and consistently supports the early detection & response to public health threats [explore enablers and barriers]	x
2.3	[Graduates] Number/percentage of CFEP graduates using outbreak management competencies [explore enablers and barriers]	x
2.4	[Graduates] Number/percentage of CFEP graduates supporting an Incident Management System (IMS) during a public health emergency response [explore enablers and barriers]	x
2.5	[Graduates] Number of outbreak investigations supported by graduates [Number/percentage of graduates supporting outbreak investigations] [explore type of outbreak / role of graduate / activities performed]	x
3.3	[Graduates] Number/percentage of graduates that feel confident to respond to emerging issues in public health	x
3.4	[Graduates] Number/percentage of graduates that feel confident in (a) outbreak management and response, (b) public health surveillance, (c) applied epidemiology, (d) scientific communication [explore enablers and barriers]	x
3.5	[Graduates] Number/percentage of graduates that feel confident to be deployed into the field for an outbreak or public health emergency response [explore enablers and barriers]	x
4.1	[Fellows] Fellows report unintended positive consequences of being a CFEP trainee while in the program [describe consequence and magnitude]	x

4.2	[Fellows] Fellows report unintended negative consequences of being a CFEP trainee while in the program [describe consequence and magnitude; were they reported to CFEP (if no, why not); were they addressed (if no, why not)]	x
4.3	[Graduates] Graduates report unintended positive consequences of being a CFEP alumni [describe consequence and magnitude]	x
4.4	[Graduates] Graduates report unintended negative consequences of being a CFEP alumni [describe consequence and magnitude; were they reported to CFEP (if no, why not); were they addressed (if no, why not)]	x
5.1	[Graduates] Number/percentage of graduates employed in epidemiology or applied public health related positions	x
5.2	[Graduates] Number/percentage of graduates placed across different tiers of health system	x
5.3	[Graduates] Graduates actively engage with and contribute to formal or informal FETP alumni network(s) [in what ways? who do you connect with? for what purpose?]	x
5.4	[Graduates] Graduates develop, engage, and use networks and partnership developed during CFEP to improve public health practice [explore types of professionals they engage with; explore what scenarios access to professional networks improved timeliness or quality of your work]	x
5.5	[Graduates] Number/percentage of graduates who are members of national, regional or international public health committees or working groups [explore types of health committees/WGs, roles]	x
6.1	Gender and ethnicity/indigenicity breakdown of graduates	x
6.2	[Fellows] Number of papers published with fellows as lead authors [Number/percentage of graduates publishing papers as lead authors, list publications]	
6.3	[Graduates] Number of papers published with graduates as lead authors [Number/percentage of graduates publishing papers as lead authors, list publications]	x
6.4	[Fellows] Number of papers published with fellows as co-authors [Number/percentage of graduates publishing papers as co-authors]	
6.5	[Graduates] Number of papers published with graduates as co-authors [Number/percentage of graduates publishing papers as co-authors]	x
6.6	Number of conference abstracts submitted by graduates being accepted [Number/percentage of graduates having conference abstracts accepted]	
6.7	Number of conference presentations given by graduates [Number/percentage of graduates giving conference presentations]	x
6.8	Graduates routinely analyse and interpret surveillance data to inform decision making	x

During the program, trainees must complete the following eight professional deliverables:

- Field investigation
- Epidemiologic analysis
- Public health surveillance system
- Peer-reviewed journal
- Public health update
- Oral presentation
- General communication
- Public health service

**CFEP Core Pillar**	**Targeted CFEP Competency Domains**
Outbreak management and response	Outbreak Investigation<br>Emergency Preparedness and Response
Public health surveillance	Surveillance<br>Data/information collection and analysis
Scientific communications	Communication
Advanced epidemiology	Data/information collection and analysis
Field Readiness	Public Health Sciences<br>Diversity and inclusiveness<br>Leadership<br>Ethics and Professionalism<br>Communication
