# Supplementary material for: Strengthening field epidemiology capacity in Canada: a mixed-methods evaluation of the Canadian field epidemiology program
Source: Front Public Health. 2026 Mar 20;14:1777134. doi: 10.3389/fpubh.2026.1777134 (PMC13047194; doi:10.3389/fpubh.2026.1777134)
Supplement: Supplementary file 2 [file Data_Sheet_2.pdf]

Evaluating the Impact of the Canadian Field Epidemiology Training Program

Graduate Interview Guide

V2.0

INTRODUCTION

Thank you for agreeing to participate in today's interview. The Public Health Agency of Canada and the University of Newcastle are working together to evaluate the Canadian Field Epidemiology Program (CFEP). We are interested in the experience of graduates to understand how the training has impacted you and your work.

The information you provide will help us understand the impact of the program and will be used to strengthen CFEP going forward.

As part of this project, I would like to ask you questions about some of your experiences following your graduation from CFEP. The interview will go for approximately 30-45 minutes.

You will have been sent an information sheet outlining the study.

Have you had the opportunity to read the information sheet?

- ☐ Yes
- ☐ No = pause interview until information sheet has been reviewed

Do you consent to this interview?

- ☐ Yes
- ☐ No = pause interview until consent has been received

A few reminders before we start:
- Your participation is voluntary; we can stop the interview at any time.
- We plan to record this interview so we can later convert it to text and analyse themes that come from all the interviews.
- The information you provide me today will be combined with the information we receive from other graduates we interview; we will summarize these findings together.
- Your name will not be linked to anything you say.

Do you have any questions for me before we start?

Are you happy if I record the interview? Recording makes it easier for me to focus on what you are saying instead of taking notes.

Consent to recording
- ☐ Yes
- ☐ No = if not, take written notes throughout interview

I am going to start recording now – *PRESS RECORD*

Questions

Thank you for agreeing to this interview.

1. Can you introduce yourself and your current role?

2. Have you been able to apply knowledge or skills learnt during your CFEP to your work?

   If no

   a. What do you think are the key reasons why you haven't been able to apply your knowledge of skills? (then go to Q3)

   If yes

   a. What knowledge and skills gained through CFEP do you most often use?

   b. What knowledge and skills gained through CFEP do you not use, or rarely use?

   c. Can you share some examples of how you have applied your CFEP knowledge or skills in you work?

      Interviewer: following the initial response, please prompt for specific examples relating to the following CFEP competencies

      Have you had the opportunity to participate in any <u>field investigations</u> since graduating? Things such as outbreak investigations, public health emergency response activities or disaster response activities.

      - Can you tell me more about that?
      - What was your role during the field investigation(s)?
      - What activities did you perform?
      - How well did CFEP prepare you for the field investigation(s)?
      - Did you develop or strengthen any field investigation tools, practices, or processes?
      - Were there any notable outcomes or impacts from your investigation? [tell me more]
      - Have you operated within an Incident Management System during an emergency response? How prepared did you feel for this?

      Have you had the opportunity to <u>collect, analyse and/or interpret health data</u>?

      - Can you tell me more about that?
      - Was that data used to make decisions? Can you give me some examples of decisions you made?
      - How well did CFEP prepare you for data collection, analysis and interpretation?
      - Did you develop or strengthen any data collection or analysis tools, practices or processes?

- Were there any notable outcomes or impacts from data collection, analysis and/or interruption? [tell me more]

Have you had the opportunity to work in the area of disease surveillance?

- Can you tell me more about that?
- How well did CFEP prepare you for disease surveillance?
- Did you develop or strengthen any disease surveillance tools, practices or processes?
- Were there any notable outcomes or impacts from your disease surveillance work? [tell me more]

Apart from what you have already mentioned, have you had the opportunity to use your CFEP knowledge and skills to make any other changes in your workplace? These changes could be things like changes to work processes, practices, policies or programs?

- Can you tell me more about that?
- What was your role in this change?
- Were there any notable outcomes or impacts from these changes? [tell me more]

3. How confident do you feel about responding to emerging public health issues?
    - How important was CFEP in building your confidence in this area?
    - Is there anything that would help you feel more prepared and confident? [tell me more]

4. What were some of the key factors that enabled or supported you to apply your knowledge and skills in your workplace? [tell me more]

5. What were some of the key barriers that prevented or inhibited you from applying your knowledge and skills in your workplace? [tell me more]

6. Is there anything that would help you apply more of your CFEP knowledge and skills, or apply them more effectively?

7. Can you share any examples of how the training had an unexpected positive impact on you personally, or on your work?

8. Can you share any examples of how the training had an unexpected negative impact on you personally, or on your work?

9. Have you used any of the networks or partnerships developed during CFEP to improve your public health practice? [tell me more]

10. Can you describe a single significant change that occurred as a result of your field epidemiology training? [you can use the same example you gave in the survey, or a different example]

    [When I refer to a significant change, I mean something you did following your training that resulted in a change in an activity, action, behaviour or practices of an individual, group or organization]

    - Can you tell me more about who was involved?
    - Can you tell me more about what changed?
    - Can you tell me about when and where this change occurred?
    - What was your contribution to this change? Were others also involved; what was their contribution?
    - Why was this change significant? How did it make a difference?

11. Do you have any recommendations for CFEP?

12. Is there anything further you would like to share regarding your CFEP training or the impact it has had on you or your work?

13. Before we conclude, we would like to interview your line-manager to obtain their perspectives regarding the outcomes of impact of CFEP on and how the trinaing has benefit their organization. Would you be willing to provide the name and contact details of your current line manager? We are only conducting interviews if they have managed you for 3 months or more. [record name, email and phone details]

Thank you for your time and input. [STOP RECORDING]

Alignment of survey questions with specific indicators

Indicator Number	Indictor	Graduate Survey Question Included
1.1	1.1. [Fellows] CFEP Field Epidemiologists were able to apply their field epidemiology knowledge and skills to benefit their placement site [explore enablers and barriers]	x
1.2	1.2. [Fellows] CFEP Field Epidemiologists were able to transfer their knowledge and skills to colleagues at their placement site [explore enablers and barriers]	Recommend survey only
1.5	1.3. [Graduates] Graduates transfer field epidemiology knowledge and skills to others through training and/or mentoring [explore enablers and barriers]	Recommend survey only
1.6	1.4. [Health system] Fellows contribute to the improvement of data collection, data management, or data analysis tools and processes. [explore enablers and barriers; explore significance of change(s); explore fellows' contribution]	x
1.7	1.5. [Health System] Number of disease surveillance systems strengthened by fellows and graduates [explore examples/categories, explore enablers and barriers; explore significance of change(s); explore fellows' contribution]	x
1.8	1.6. [Health System] Number of outbreak response tools and practices strengthened by fellows and graduates [explore examples/categories, explore enablers and barriers; explore significance of change(s); explore fellows' contribution]	x
1.9	1.7. [Health System] Graduates contribute to strong disease surveillance systems that guide public health programming and consistently supports the early detection & response to public health threats [explore enablers and barriers]	x
2.1	2.1. [Graduates] Number/percentage of CFEP graduates using outbreak management competencies [explore enablers and barriers]	Recommend survey only
2.2	2.2. [Graduates] Number/percentage of CFEP graduates supporting an Incident Management System (IMS) during a public health emergency response [explore enablers and barriers]	x
2.3	2.3. [Graduates] Number of outbreak investigations supported by graduates [Number/percentage of graduates supporting outbreak investigations] [explore type of outbreak / role of graduate / activities performed]	x

3.1	3.1. [Graduates] Number/percentage of graduates that feel confident to respond to emerging issues in public health	x
3.2	3.2. [Graduates] Number/percentage of graduates that feel confident in (a) outbreak management and response, (b) public health surveillance, (c) applied epidemiology, (d) scientific communication [explore enablers and barriers]	Recommend survey only
3.3	3.3. [Graduates] Number/percentage of graduates that feel confident to be deployed into the field for an outbreak or public health emergency response [explore enablers and barriers]	x
4.1	4.1. [Fellows] Fellows report unintended positive consequences of being a CFEP trainee while in the program [describe consequence and magnitude]	x
4.2	4.2. [Fellows] Fellows report unintended negative consequences of being a CFEP trainee while in the program [describe consequence and magnitude; were they reported to CFEP (if no, why not); were they addressed (if no, why not)]	x
4.3	4.3. [Graduates] Graduates report unintended positive consequences of being a CFEP alumni [describe consequence and magnitude]	x
4.4	4.4. [Graduates] Graduates report unintended negative consequences of being a CFEP alumni [describe consequence and magnitude; were they reported to CFEP (if no, why not); were they addressed (if no, why not)]	x
5.1	5.1. [Graduates] Graduates actively engage with and contribute to formal or informal FETP alumni network(s) [in what ways? who do you connect with? for what purpose?]	Recommend survey only
5.2	5.2. [Graduates] Graduates develop, engage, and use networks and partnership developed during CFEP to improve public health practice [explore types of professionals they engage with; explore what scenarios access to professional networks improved timeliness or quality of your work]	x
5.3	5.3. [Graduates] Number/percentage of graduates who are members of national, regional or international public health committees or working groups [explore types of health committees/WGs, roles]	Recommend survey only
6.1	6.1. Graduates are developing or contributing to the development of public health policy [which policies? what was contribution? outcome?]	x
6.2	6.2. Graduates are introducing improved ways of delivering public health programs [which programs? what was contribution? outcome?]	x

NOTES:

During the program, trainees must complete the following eight professional deliverables:

- Field investigation
- Epidemiologic analysis
- Public health surveillance system
- Peer-reviewed journal
- Public health update
- Oral presentation
- General communication
- Public health service

**CFEP Core Pillar**	**Targeted CFEP Competency Domains**
Outbreak management and response	Outbreak Investigation<br>Emergency Preparedness and Response
Public health surveillance	Surveillance<br>Data/information collection and analysis
Scientific communications	Communication
Advanced epidemiology	Data/information collection and analysis
Field Readiness	Public Health Sciences<br>Diversity and inclusiveness<br>Leadership<br>Ethics and Professionalism<br>Communication

Evaluating the Impact of the Canadian Field Epidemiology Training Program

Placement Supervisor Interview Guide

V2.0

INTRODUCTION

Thank you for agreeing to participate in today's interview. The Public Health Agency of Canada and the University of Newcastle are working together to evaluate the Canadian Field Epidemiology Program (CFEP). We are interested in the experience and reflections of CFEP placement supervisors and their feedback on the training program and the graduates under their supervision.

The information you provide will help us understand the impact of the program and will be used to strengthen CFEP going forward.

The interview will go for approximately 20-30 minutes

Can I confirm that you were the placement supervisor of [Name of Graduate]?

- ☐ Yes
- ☐ No = stop interview

You will have been sent an information sheet outlining the study. Have you had the opportunity to read the information sheet?

- ☐ Yes
- ☐ No = pause interview until information sheet has been reviewed

Do you consent to this interview?

- ☐ Yes
- ☐ No = pause interview until consent has been received

A few reminders before we start:

- Your participation is voluntary; we can stop the interview at any time.
- We plan to record this interview so we can later convert it to text and analyse themes that come from all the interviews.
- The information you provide me today will be combined with the information we receive from other graduates we interview; we will summarize these findings together.
- Your name will not be linked to anything you say.

Do you have any questions for me before we start?

Are you happy if I record the interview? Recording makes it easier for me to focus on what you are saying instead of taking notes.

Consent to recording

- ☐ Yes
- ☐ No = if not, take written notes throughout interview

I am going to start recording now – *PRESS RECORD*

Questions

Thank you for agreeing to this interview.

1. Can you please introduce yourself and your current role?

2. Was [Graduate] able to apply his/her field epidemiology knowledge and skills in a way that was beneficial to you and the placement site?
   - What were the most valuable skills [Graduate] brought to your workplace?

3. Can you share any examples of how [Graduate] contributed to public health activities in the workplace?

   Interviewer: following the initial response, please prompt for specific examples relating to the following CFEP competencies

   - Did [Graduate] contribute to a field investigation such as an outbreak, emergency response, or disaster response?
     - What type of investigation(s) did they respond to?
     - What was their role on the investigation team(s)?
     - Did [Graduate] strengthen any field investigation tools, practices, or processes? [tell me more]
     - Were there any notable outcomes or impacts from their investigation(s)? [tell me more]

   - Did [Graduate] contribute to data collection, analysis and/or interpretation?
     - Was the data collected/analysed used to guide decision making? If so, what decisions were made and by whom?
     - Did [Graduate] improve any data collection, management or analysis tools, practices, or processes? [tell me more]
     - Were there any notable outcomes or impacts arising from the data collection, analysis and/or interpretation they were involved with? [tell me more]

   - Did [Graduate] contribute to any disease surveillance activities?
     - What was his/her role in disease surveillance?
     - Did he/she evaluate a surveillance system and make recommendations for strengthening that system? [tell me more]
     - Were there any notable outcomes or impacts of their disease surveillance activities? [tell me more]

- Did [Graduate] contribute to changing a health program or policy?
    - Can you tell me about that change? [probe for both health program and policy change]
    - What was their role in bringing about this change/these changes?
    - Were there any notable outcomes or impacts from their work on changing a health program or policy? [tell me more]

- Did [Graduate] contribute to strengthening or changing any other workplace process or practice that you are aware of?
    - Can you tell me about that change?
    - What was their role in bringing about this change/these changes?
    - Were there any notable outcomes or impacts from their work on changing workplace processes or practices? [tell me more]

4. Were there any barriers that limited [Graduate's] ability to apply his/her field epidemiology knowledge and skills in the workplace?
    - Is there anything that would have supported [Graduate] apply more skills or apply them in a more effective way?

5. While placed with you, did [Graduate] share his/her field epidemiology knowledge and skills with others in the workplace? This could have been through training, mentoring, or sharing new techniques, tools or approaches with others.
    - In what ways did he/she share their knowledge/skills?
    - Approximately how many individuals were supported or impacted by [Graduate]

6. Is there anything further you would like to share regarding CFEP, the field epidemiologists you have supervised, or the impact it has had or not had in the workplace?

Thank you for your time and input. [STOP RECORDING]

Alignment of survey questions with specific indicators

Indicator Number	Indictor	Placement Supervision Question Included
1.1	[Fellows] CFEP Field Epidemiologists were able to apply their field epidemiology knowledge and skills to benefit their placement site [explore enablers and barriers]	X
1.2	[Fellows] CFEP Field Epidemiologists were able to transfer their knowledge and skills to colleagues at their placement site [explore enablers and barriers]	X
1.4	[Fellows] Number/percentage of surveillance system evaluations where one or more of the recommendations made by the Field Epidemiologist have been implemented with one year of the evaluation [explore enablers and barriers; explore significance of change(s); explore fellows' contribution]	X
1.6	[Health system] Fellows contribute to the improvement of data collection, data management, or data analysis tools and processes. [explore enablers and barriers; explore significance of change(s); explore fellows' contribution]	X
1.7	[Health System] Number of disease surveillance systems strengthened by fellows and graduates [explore examples/categories, explore enablers and barriers; explore significance of change(s); explore fellows' contribution]	X
1.8	[Health System] Number of outbreak response tools and practices strengthened by fellows and graduates [explore examples/categories, explore enablers and barriers; explore significance of change(s); explore fellows' contribution]	X

Evaluating the Impact of the Canadian Field Epidemiology Training Program

Current Manager Interview Guide

V2.0

INTRODUCTION

Thank you for agreeing to participate in today's interview. The Public Health Agency of Canada and the University of Newcastle are working together to evaluate the Canadian Field Epidemiology Program (CFEP). We are interested in your experience and reflections as a manager of a CFEP graduate.

The information you provide will help us understand the impact of the program and will be used to strengthen CFEP going forward.

The interview will go for approximately 20-30 minutes

Can I confirm that you are the manager of [Name of Graduate], and have been his/her manager for 3 or more months?

- ☐ Yes
- ☐ No = stop interview

You will have been sent an information sheet outlining the study. Have you had the opportunity to read the information sheet?

- ☐ Yes
- ☐ No = pause interview until information sheet has been reviewed

Do you consent to this interview?

- ☐ Yes
- ☐ No = pause interview until consent has been received

A few reminders before we start:

- Your participation is voluntary; we can stop the interview at any time.
- We plan to record this interview so we can later convert it to text and analyse themes that come from all the interviews.
- The information you provide me today will be combined with the information we receive from other graduates we interview; we will summarize these findings together.
- Your name will not be linked to anything you say.

Do you have any questions for me before we start?

Are you happy if I record the interview? Recording makes it easier for me to focus on what you are saying instead of taking notes.

Consent to recording

- ☐ Yes
- ☐ No = if not, take written notes throughout interview

I am going to start recording now – *PRESS RECORD*

Questions

Thank you for agreeing to this interview.

1. Can you please introduce yourself and your current role?

2. How long have you been the manager of [Graduate]?

3. Has [Graduate] been able to apply his/her field epidemiology knowledge and skills in a way that has been beneficial to you and your team?
   - What are the most valuable skills [Graduate] has brought to your workplace?

4. Can you share any examples of how [Graduate] has contributed to public health activities in the workplace?

   Interviewer: following the initial response, please prompt for specific examples relating to the following CFEP competencies

   - Has [Graduate] contributed to a field investigation such as an outbreak, emergency response, or disaster response?
     - What type of investigation(s) did they respond to?
     - What was their role on the investigation team(s)?
     - Has [Graduate] strengthened any field investigation tools, practices, or processes? [tell me more]
     - Have there been any notable outcomes or impacts from their investigation(s)? [tell me more]
     - Are there any aspects of a field investigation that you are not confident [Gradate] could perform?

   - Has [Graduate] contributed to data collection, analysis and/or interpretation?
     - Was the data collected/analysed used to guide decision making? If so, what decisions were made and by whom?
     - Has [Graduate] improved any data collection, management or analysis tools, practices, or processes? [tell me more]
     - Have there been any notable outcomes or impacts arising from the data collection, analysis and/or interpretation they have been involved in? [tell me more]

    - Are there any aspects of data collection, analysis and/or interpretation that you are not confident [Gradate] could perform?

- Has [Graduate] contributed to any <u>disease surveillance</u> activities?
    - What was or is his/her role in disease surveillance?
    - Has he/she evaluated a surveillance system and made recommendations for strengthening that system? [tell me more]
    - Have there been any notable outcomes or impacts of their disease surveillance activities? [tell me more]
    - Are there any aspects of disease surveillance that you are not confident [Gradate] could perform?

- Has [Graduate] contributed to changing a <u>health program or policy</u>?
    - Can you tell me about that change? [probe for both health program and policy change]
    - What was his/her role in bringing about this change/these changes?
    - Have there been any notable outcomes or impacts from their work on changing a health program or policy? [tell me more]

- Has [Graduate] contributed to strengthening or changing any <u>other workplace processes or practices</u> that you are aware of?
    - Can you tell me about that change?
    - What was their role in bringing about this change/these changes?
    - Have there been any notable outcomes or impacts from their work on changing workplace processes or practices? [tell me more]

5. Have there been, or are there currently, any barriers that limits [Graduate's] ability to applying his/her field epidemiology knowledge and skills in the workplace?
    - Is there anything that would support [Graduate] apply more skills or apply them in a more effective way?

6. Is there anything further you would like to share regarding CFEP, the fellows you have supervised, or the impact it has had or not had in the workplace?

Thank you for your time and input. [STOP RECORDING]

[Interviewer: as appropriate, please seek input on the graduate's outcome statement(s) after you conclude the interview]

7. Has [Graduate] been able to share his/her field epidemiology knowledge and skills with others in the workplace? This could have been through training, mentoring, or sharing new techniques, tools or approaches with others.

   [Prompt]

   - If yes, approximately how many individuals were supported or impacted by [Graduate]

8. Can you share any examples of how [Graduate] has contributed to public health activities in the workplace?

   [Prompts]

   - Has [Graduate] contributed to a field investigation?
     - What type of investigation(s) has [Graduate] identified and/or responded to? What was their role on the investigation team?
     - Has [Graduate] strengthened any field investigation tools, practices or processes?
     - Are there any significant outcomes or impacts associated with [Graduates] field investigation work? Are these outcomes/impacts ongoing?
     - Are there any aspects of a field investigation that you are not confident with [Gradate] performing?
     - Are there any other barriers to [Graduate] supporting or leading field investigation activities?

   - Has [Graduate] contributed to data collection, analysis and/or interpretation related activities?
     - Was the data used to make any decisions? What decisions were made and who made them?
     - Has [Graduate] improved any data collection, management or analysis tools, practices or processes?
     - Have there been any significant outcomes or impacts associated with [Graduates] work in data collection, analysis and interpretation? Are these outcomes/impacts ongoing?

   - Has [Graduate] contributed to any disease surveillance activities?
     - What is his/her role in disease surveillance?
     - Has [Graduate] made any changes to strengthen a surveillance system? What was [Graduates] contribution to this change?

    - Does [Graduate] routinely analyse and/or interpret surveillance data to inform decision making?

- Has [Graduate] contributed to changing a health program or policy?
    - Can you tell us about that change? [probe for both health program and policy]
    - Have there been any significant outcomes or impacts associated with [Graduates] work in this area? Are these outcomes/impacts ongoing?

- Has [Graduate] contributed to strengthening or changing any other workplace process or practice that you are aware of?
    - Can you tell us about that change in process or practice?
    - What were the results of that change?
    - Have there been any significant outcomes or impacts associated with [Graduates] work in this area? Are these outcomes/impacts ongoing?

9. Is there anything further you would like to share regarding CFEP, its graduates and the impact it has had in the workplace?

Thank you for your time and input. [STOP RECORDING]

Alignment of survey questions with specific indicators

Indicator Number	Indictor	Work Manager Question Included
1.5	[Graduates] Graduates transfer field epidemiology knowledge and skills to others through training and/or mentoring [explore enablers and barriers]	X
1.7	[Health System] Number of disease surveillance systems strengthened by fellows and graduates [explore examples/categories, explore enablers and barriers; explore significance of change(s); explore fellows' contribution]	X
1.8	[Health System] Number of outbreak response tools and practices strengthened by fellows and graduates [explore examples/categories, explore enablers and barriers; explore significance of change(s); explore fellows' contribution]	X
1.9	[Health System] Graduates contribute to strong disease surveillance systems that guide public health programming and consistently supports the early detection & response to public health threats [explore enablers and barriers]	X
2.3	[Graduates] Number/percentage of CFEP graduates using outbreak management competencies [explore enablers and barriers]	X
6.8	Graduates routinely analyse and interpret surveillance data to inform decision making	X
6.11	Graduates are recognized, utilized and have influence in the workplace	X
