# Supplementary material for: Strengthening field epidemiology capacity in Canada: a mixed-methods evaluation of the Canadian field epidemiology program
Source: Front Public Health. 2026 Mar 20;14:1777134. doi: 10.3389/fpubh.2026.1777134 (PMC13047194; doi:10.3389/fpubh.2026.1777134)
Supplement: Supplementary file 3 [file Supplementary_File_1.docx]

**Supplementary Table 1.** Type of communicable or non-communicable disease outbreak responded to by CFEP graduates during their training, 2018-2023.

| Graduate (year) | COVID-19 | Enteric disease | Vaccine preventable disease | iGAS | Tuberculosis | HIV | STI | Drugs | mPox | Legionella | Non-communicable disease | Acute respiratory illness | Blood-borne disease | Other |
| --- | --- | --- | --- | --- | --- | --- | --- | --- | --- | --- | --- | --- | --- | --- |
| 1-2018 |  |  | x |  |  | x |  | x |  |  |  |  |  |  |
| 2-2018 |  |  |  | x |  |  |  |  |  |  |  |  |  | x |
| 3-2018 |  | x | x |  |  | x |  |  |  |  |  |  |  |  |
| 4-2018 |  |  |  | x |  |  |  |  |  |  |  |  |  |  |
| 5-2019 |  | x |  |  | x |  |  |  |  |  |  |  |  |  |
| 6-2019 |  |  |  |  | x |  |  |  |  |  |  |  |  |  |
| 7-2020 | x | x | x | x |  |  |  |  |  |  |  |  |  | x |
| 8-2020 | x |  |  |  |  |  | x |  |  |  |  |  | x |  |
| 9-2020 | x | x | x |  |  |  |  |  |  |  |  |  |  |  |
| 10-2020 | x | x |  |  | x |  | x |  |  |  |  |  |  |  |
| 11-2020 | x |  |  |  |  |  |  | x |  |  | x |  |  |  |
| 12-2021 | x |  |  |  |  |  |  |  |  |  |  |  |  |  |
| 13-2021 | x | x |  |  |  |  |  |  |  |  |  |  |  |  |
| 14-2021 | x | x |  |  |  |  |  |  |  |  |  |  |  | x |
| 15-2021 | x |  |  |  |  |  |  |  |  |  |  |  |  |  |
| 16-2022 | x |  | x |  |  |  |  |  | x |  |  | x |  |  |
| 17-2022 | x |  |  | x |  |  |  |  |  | x |  |  |  |  |
| 18-2022 | x | x |  |  |  |  |  |  |  |  |  |  |  |  |
| 19-2023 |  | x |  |  |  |  |  |  |  |  |  |  |  |  |
| 20-2023 |  | x |  |  |  |  |  |  |  | x |  |  |  |  |
| 21-2023 | x | x |  | x | x |  |  |  | x |  |  |  |  |  |
| **Total** | **13** | **11** | **5** | **5** | **4** | **2** | **2** | **2** | **2** | **2** | **1** | **1** | **1** | **3** |
| **Percent** | **62%** | **52%** | **24%** | **24%** | **19%** | **10%** | **10%** | **10%** | **10%** | **10%** | **5%** | **5%** | **5%** | **14%** |

**Supplementary Table 2.** Outbreaks investigated by 2018-2023 graduates following CFEP, Canada, 2024.

| **Graduation year** | **Description of outbreak** |
| --- | --- |
| 2018 | COVID-19, Mumps in Ontario |
| 2018 | COVID-19, Monkeypox |
| 2018 | Tuberculosis in Nunatsiavut, COVID-19 in various places in Newfoundland and Labrador |
| 2018 | COVID-19 Trenton base |
| 2019 | COVID-19 |
| 2020 | COVID-19 outbreaks in Montérégie |
| 2020 | COVID-19 outbreak in a school in Ontario |
| 2020 | COVID-19 outbreak in an industrial setting in British Columbia (lead investigator); Several COVID-19 variant introductions into Canada (team-lead); Sexually Transmitted and Blood Borne Infection outbreak in Ontario |
| 2020 | COVID-19 (Supervisory and manager roles at national level); Supervisor of the FluWatch team; manager of the Data Integration team which developed the national COVID-19 case database; manager of the Data Analytics team for the National Emergency Strategic Stockpile |
| 2020 | COVID-19 |
| 2020 | COVID-19 |
| 2020 | Wildfire Emergency Response; liaison for our Centre’s response |
| 2020 | COVID-19 |
| 2021 | Foodborne Disease outbreaks (lead investigator) at the national level (outbreaks include E. coli in kimchi, Salmonella in cantaloupes, norovirus in oysters, norovirus in spot prawns, and Cyclospora) |
| 2021 | Disaster response (Grade 3 Food and Drought Emergency in the Greater Horn of Africa) |
| 2022 | Foodborne Disease outbreaks (National multi-jurisdictional outbreaks) |
| 2022 | Syphilis outbreak in British Columbia |
